# Supplementary material for: Borate driven heterogeneous networks for porous elastomers with improved tribological and mechanical performances
Source: Nat Commun. 2025 Dec 17;16:11183. doi: 10.1038/s41467-025-66156-2 (PMC12712056; doi:10.1038/s41467-025-66156-2)
Supplement: Supplementary file 1 — Supplementary Information [file 41467_2025_66156_MOESM1_ESM.pdf]

Supplementary Information

Borate Driven Heterogeneous Networks for Porous Elastomers with Improved Tribological and Mechanical Performances

Yuhao Wu [1], Liguo Qin [1]*, Zeyu Ma [1], Mingqing Sun [1], Zheng Wang [1], Shan Lu [1], Xiaodong Huang [1], Wentao Xia [1], Hao Yang [1], Jianbo Liu [1], Ke Yan [1], Xin Ge [2]*, Sen Yang [3]*, and Guangneng Dong [1]

[1]Key Laboratory of Education Ministry for Modern Design and Rotor-Bearing System, Institute of Design Science and Basic Components, School of Mechanical Engineering, Xi'an Jiaotong University, Xi'an 710049, P. R. China.

[2]Department of Materials-Oriented Chemical Engineering, School of Chemical Engineering, Fuzhou University, Fuzhou 350116, P. R. China.

[3]School of Physics, MOE Key Laboratory for Nonequilibrium Synthesis and Modulation of Condensed Matter, Xi'an Jiaotong University, Xi'an, 710049 P. R. China.

*Corresponding author:

Email: liguoqin@xjtu.edu.cn (Liguo Qin), gexin@fzu.edu.cn (Xin Ge), yangsen@mail.xjtu.edu.cn (Sen Yang).

This PDF file includes:

1. Supplementary Tables: Supplementary Tables 1-4

2. Supplementary Discussion

3. Supplementary Figures: Figs. 1-57

4. Supplementary References

1. Supplementary Tables

Supplementary Table 1. Abbreviation descriptions for different types of elastomers

Type	Methods or materials	Abbreviation
Polydimethylsiloxane	Pure polydimethylsiloxane	PDMS
	Sylgard 184A+Sylgard 184B	S-PDMS
	Vinyl-terminated polydimethylsiloxane (main content of Sylgard 184A)	VPDMS
	Silicon-hydrogen bonded polydimethylsiloxane (main content of Sylgard 184B)	SHPDMS
	Hydroxyl-terminated polydimethylsiloxane	HPDMS
Boric acid and its derivatives	Boric acid	BA
	Methylboric acid	MBA
	Phenylboric acid	PBA
	Phenyldiboric acid	PdBA
	Biphenyl diboric acid	bPdBA
Dynamic crosslinking PDMS with boric acid ester	Dynamic crosslinking PDMS synthesized with BA, MBA, PBA, PdBA, or bPdBA as the crosslinking agent	D-PDMS@BA, D-PDMS@MBA, D-PDMS@PBA, D-PDMS@PdBA, D-PDMS@bPdBA
Heterogeneous-network PDMS	Heterogeneous-network PDMS formed by D-PDMS with a BA ester or bPdBA ester and S-PDMS interpenetrating	HN-PDMS@BA, HN-PDMS@bPdBA
Heterogeneous-network porous PDMS	Some D-PDMS (D-PDMS@BA, D-PDMS@MBA, D-PDMS@PBA, D-PDMS@PdBA, or D-PDMS@bPdBA) in the heterogeneous-network PDMS is removed	HNP-PDMS@BA, HNP-PDMS@MBA, HNP-PDMS@PBA, HNP-PDMS@PdBA, or HNP-PDMS@bPdBA

Porous PDMS	Sugar template method<br>NaCl template method<br>Glycerin emulsion method<br>Castor oil emulsion method<br>$NaHCO_3$ foaming method	P-PDMS@sugar<br>P-PDMS@NaCl<br>P-PDMS@Gly<br>P-PDMS@castor<br>P-PDMS@$NaHCO_3$
Other silicone	Ecoflex 0031<br>Sylgard 170<br>Sylgard 527<br>Momentive RTV615	ES<br>DC170<br>DC527<br>RTV615
Other Porous silicone/PDMS	Some D-PDMS@BA in the heterogeneous silicone/PDMS network is removed	ES@BA<br>DC170@BA<br>DC527@BA<br>RTV615@BA
Heterogeneous-network porous PDMS with carbon fibers	Some D-PDMS@BA in the heterogeneous-network PDMS with carbon fibers is removed	HNP-PDMS@C

Elastomers are denoted as X@Y-A/B/C, where *X* represents the type of PDMS, *Y* denotes the crosslinking agent, *A* indicates the curing temperature, *B* specifies the curing pressure, and *C* represents the mass percentage of dynamic crosslinking PDMS. If *C* is absent, the default mass percentage is 20%. **Especially, P1 and P2 represent HNP-PDMS@BA-125/-0.1MPa and HNP-PDMS@BA-125/0MPa in the friction performance section.**

Supplementary Table 2. The value of cohesive energy density and solubility parameter.

	Cohesive energy density (J/m^3)	Solubility parameter (J/cm^3) ^ 0.5
PDMS+PB	9.003e+007	9.488
PDMS	1.067e+008	10.331
PB	9.078e+007	9.523

Supplementary Table 3. Diffusion constants of B atoms in the system of B.

Temperature (K)	Pressure (MPa)	D (cm^2/s)
398	0	4.07×10^{-6}
300	0	3.16×10^{-6}
300	0.1	2.28×10^{-6}

Supplementary Table 4. The 12-6 Lennard–Jones potential parameters for solid particle and water particle.

Type	ε (kcal/mol)	σ (Å)
Ball- Water	0.43	2.582
Water- Surface (hydrophobic)	0.33	2.582
Water- Surface (hydrophilic)	0.48	2.582

2. Supplementary Discussion

2.1 Supplementary phase separation

Based on the wavelengths (ν) of B-O and Si-O displayed by FTIR, the force constant (k) can be calculated according to the following formula:

$$\nu = \frac{1}{2\pi c}\sqrt{\frac{k}{\mu}} \tag{1}$$

where c and μ are speed of light and reduced mass. Because of k_{B-O} (677 N/m)> k_{Si-O} (606 N/m), bond strength of B-O is more than that of Si-O. Then, we evaluated bond dissociation energies (BDEs) for two chemically distinct bonds within the target molecule, Si–O (siloxane bridge) and B–O (the terminal B–O–Si linkage). To avoid open-shell radicals and ensure closed-shell fragments (Frag), we employed a hydrogenation (H_2-capping) scheme that conserves atom counts:

For a generic A–B bond in the parent molecule M, define the reaction:

$$M + H_2 \rightarrow Frag(A-H) + Frag(B-H),$$

where each fragment is constructed by cleaving A–B and adding one H atom to A and one H atom to B to satisfy valence. The bond dissociation energy is then

$$EBDE(A-B) = E[Frag(A-H)] + E[Frag(B-H)] - E[M] - E[H_2] \tag{2}$$

(i) Si–O (siloxane) bond inside the chain)

Cut the selected Si–O bridge in HO- [Si $(CH_3)_2$–O-]$_6$–$B(OH)_2$, yielding two fragments: the Si-terminated part is capped to –Si $(CH_3)_2$–H, and the O-terminated part is capped to –O–H (silanol). Apply Eq. (S2):

$$EBDE(Si-O) = E[Frag\ with\ Si-H] + E[Frag\ with\ O-H] - E[M] - E[H_2] \tag{3}$$

(ii) B–O (terminal B–O–Si linkage)

Cleaving the B–O bond that connects $B(OH)_2$ to the chain gives (a) the chain end capped to –Si–O–H and (b) a boron fragment $HB(OH)_2$ (i.e., B gains one H). The corresponding BDE is

$$EBDE(B-O) = E[Si-O-H\ terminated\ chain] + E[HB(OH)_2] - E[M] - E[H_2] \tag{4}$$

This H_2-capping protocol yields closed-shell fragments and is robust for siloxane and boron–oxygen bonds. For nonequivalent Si–O bonds along the chain (e.g., near termini vs. interior), Eqs. S3 and 4 were applied to each distinct local environment.

Based on the above method, the calculation results by density functional theory show that the BDEs of B-O and Si-O are 6.05 eV and 5.65 eV (BDE_{B-O}> BDE_{Si-O}), respectively. The BDE results are consistent with the force stiffness results. Based on the above molecular model (HO- [Si $(CH_3)_2$–O-]$_6$–$B(OH)_2$), we calculated the electrostatic potential of the molecule as shown in the Supplementary Fig. 3 to intuitively display interactions of bonds with the siloxane backbone. When boric acid and HPDMS are cross-linked, a distinct repulsive region is formed. From the electrical potential values, compared with the other groups, the silanol group is more likely to be closer to the boric acid; The non-polar methyl groups and polar hydroxyl groups are less likely to attract each other. To investigate the dynamic reversibility, we performed FTIR tests on D-PDMS@BA ethanol solution and D-PDMS@BA after evaporation of ethanol. FTIR result in Supplementary Fig. 2B displays that the B-O and C-O peaks of D-PDMS dissolved in ethanol were broadened and shifted, respectively. The FTIR result of D-PDMS after evaporation of ethanol are consistent with that of D-PDMS not dissolved in ethanol. These results illustrate the dynamic reversibility of D-PDMS under the influence of ethanol.

The first network consists of a D-PDMS, while the second network is derived from Sylgard 184 PDMS components (VPDMS, SHPDMS, and platinum catalyst). Containing BA crosslinking agent needs to be dissolved in ethanol during the preparation of D-PDMS, and HNP-PDMS obtained also needs to be processed via ethanol. These processions all follow as alcoholysis reactions as shown in Supplementary Fig. 5. Besides, a part of SHPDMS can react with the HPDMS facilitated by a platinum-based catalyst, which also promotes the entanglement of the D-PDMS in the continuous phase of the HNP-PDMS (Supplementary Fig. 6). According to the previous study[1], boroxine will be generated after the reaction between PDMS and boric acid. The presence of boroxine is verfied by the result of FTIR as shown in Supplementary Fig. 2A. Boroxanes with multiple boronate esters support the speculation of B atom enriched at the two-phase boundary.

For the MD simulations in the main text, we followed the following procedure. Mixing energy and Flory-Huggins parameter are important indicators to characterize the compatibility of copolymers, so we calculated according to the following formula:

$$\frac{\Delta E_{mix}}{V} = \phi_A \left(\frac{E_{coh}}{V}\right)_A + \phi_B \left(\frac{E_{coh}}{V}\right)_B - \left(\frac{E_{coh}}{V}\right)_{AB} \tag{5}$$

where ΔE_{mix}, V, ϕ_A ϕ_B, $\left(\frac{E_{coh}}{V}\right)_A$, $\left(\frac{E_{coh}}{V}\right)_B$, $\left(\frac{E_{coh}}{V}\right)_{AB}$ are mixing energy, volume, the volume fraction of A, B, the cohesive energy density (CED) of A ,B, A and B mixtures.

$$X = \frac{1}{\phi_A \phi_B}\left(\frac{\Delta E_{mix}}{k_B T}\right) \quad (6)$$

where χ, k_B, T are Flory-Huggins parameter, Boltzmann constant, Kelvin degree.

The radial distribution function (RDF) can be interpreted as the ratio of the local density to the global density of the system, which reflects the change of particle density with distance. It follows the following equation:

$$\mathrm{g(r)} = 4\frac{dN}{\rho 4\pi r^2 dr} \quad (7)$$

where, dN is the number of molecules in the region of $r \rightarrow r + dr$. RDF is used to describe the probability of finding an atom within a specific distance range.

Mean square displacement (MSD) is a time-dependent physical quantity that reflects the motion of particles in a system. It is defined as follows:

$$\mathrm{MSD} = \frac{1}{N}\sum_{i=1}^{N} |x_i(t_0 + t) - x_i(t_0)|^2 \quad (8)$$

where, N, t, x_i are respectively number of particles, time, the position vector of the particle. When t is large, MSD approximates a straight line. According to Einstein's diffusion law, diffusion constant (D) follows[2]:

$$\mathrm{D} = \frac{dMSD}{6dt} \quad (9)$$

The 2D SAXS patterns were averaged by symmetric sector scanning and azimuthal scanning to plot the scattering intensity against the scattering vector q, for further analysis based on Guinier's law[3], and Porod law[4].The Guinier law was used to evaluate the mean-square radius of gyration. The logarithm of the 1D SAXS profile in the perpendicular direction $ln[I(q)]$was plotted against q^2. From the slope of the linear portion in the low q region, the change of cross-sectional gyration radius (R_g) could be estimated based on the following equation:

$$ln[I(q)] = ln[I(0)] - \frac{R_g^2 q^2}{3} \quad (10)$$

The Porod-Debye function noted that the scattering data transformed as $q^2 \cdot I(q)$ against q shows in Supplementary Fig. 8, which should capture a defined area as the Porod invariant Q defined as[5]:

$$Q = \int_0^{\infty} q^2 \cdot I(q) dq = c \cdot 2\pi^2 (\triangle \rho)^2 \cdot V \quad (11)$$

The determination of Q could be used to determine several molecule structural parameters like particle's volume, surface-to-volume ratio and correlation length.[6]

$$V = 2\pi^2 \frac{I(0)}{Q} \tag{12}$$

$$\frac{S}{V} = \pi \cdot \lim_{q \to 0} \frac{I(q)q^4}{Q} \tag{13}$$

The Porod's law points out that in the range of large q or high angle, if there is a sharp interface between the two phases, the product of the scattering intensity $I(q)$ and q^3 tends to a certain constant K, which can be expressed as:

$$\lim_{q \to \infty} ln[q^3 I(q)] = ln\, K \tag{14}$$

Figure 3b1 is the Porod curve of the initial sample obtained according to the scattering curve.

2.2 Supplementary morphology and porous structure induced by network sacrificing

We used the following formula to calculate the mass percentage of the lost material to characterize whether all the D-PDMS@BA is dissolved:

$$\Delta\, m = m_1 - m_2 \tag{15}$$

where $\Delta\, m$, m_1, m_2 are respectively the mass of dissolved D-PDMS@BA, the original mass of HN-PDMS@BA, the mass of D-PDMS@BA. We use the following formula to calculate the absorption of HNP-PDMS@BA:

$$\gamma = \frac{m_o - m_2}{m_2} \times 100\% \tag{16}$$

where γ, m_o are respectively oil saturated absorption capacity, the volume of silicone oil and HNP-PDMS@BA, the density of silicone oil, the mass of HNP-PDMS@BA filled with silicone oil respectively. The γ in the Fig. 4E is the average of 3 different samples cured under the same conditions. Roundness (θ) was used to quantify the irregularity of pores,

$$\theta = \frac{r_{min}}{r_{max}} \tag{17}$$

where r_{min}, r_{max} are the minimum and the max of radium. The roughness parameters are valid for a rectangular sampling area (A) with the lateral directions x and y and vertical direction z. Sq defines the root mean square value of ordinate values within the definition area. It is equivalent to the standard deviation of heights. The roughness parameter used in this study are described below[7]:

$$Sq = (\frac{1}{A} \iint_A |Z^2(x,y)| dxdy)^{\frac{1}{2}} \tag{18}$$

Sq also describes the degree of surface porosity from another perspective. Three 3D topographic surfaces were fabricated by an optical surface metrology confocal profilometer. The Sq in the Fig. 4F is the average of 3 different samples cured under the same conditions.

2.3 Supplementary Friction Performance

Wear volume generally continues to increase with time. Wear volume is calculated based on the product of sliding distance and wear scar cross-sectional area. The sliding distance follows as:

$$X = 0.002 \times t \times f \times L \quad (19)$$

$$N = t \times f \quad (20)$$

where:

X = total sliding distance of the ball, m,

N = number of cycles in the test,

t = test time, s,

f = oscillating frequency, Hz (cycles/s), and

L = length of stroke, mm.

To investigate the stability of the low-friction transition and low-wear state, we perform fatigue and aging tests on the P1 (HNP-PDMS@BA-125/-0.1MPa). As shown in Supplementary Fig. 33, with reciprocating cycles progressing and water evaporating, the COF increases, but the COF quickly decreases to a lower level after adding a little (~0.1 mL) salt water. SEM and EDS results show that the pores of P1 are always able to capture the wear debris during this process (Supplementary Fig. 34A). COF results of other tests are shown in Supplementary Fig. 35. Friction testing of the sample after cyclic stretching (over 2000 cycles, with a stretch ratio of 20% in each cycle) revealed that it still exhibited low friction, with the average COF less than 0.15. Furthermore, after 3 weeks of salt water immersion, UV irradiation (365 nm), and heating treatment (60 °C), P1 can stably reduce the COF. An increase in the COF at 60 °C after 10000 cycles, which is due to the evaporation of water. However, the low COF state can be recovered after adding salt water. In any case, the wear rate of the aging sample (< 6.69×10^{-5} $mm^3/N\cdot m$) was reduced by over 75% compared with the wear rate of S-PDMS-G, as shown in Supplementary Fig. 36. In particular, when the friction time was extended to 8.5 h (122400 cycles), the wear rate was further reduced to as low as

~8.62×10^{-6} mm^3/N·m. We repeated the friction test of P1-G three times in situ without processing and found that COF reduction still occurred (Supplementary Fig. 37). After repeated friction tests, the CA is still close to that after the first friction test (Supplementary Fig. 23C and D). The corresponding SEM and EDS results of the worn surface are shown in Supplementary Fig. 34B, which reveals the presence of wear debris and iron in the pores, indicating that the nanoclusters were still trapped. To study the effect of product detachment on friction, after the first friction test, the mixed liquid on the surface was wiped off by the dust-free fabric and the new friction test was carried out. The COF dropped rapidly with almost no running-in period of thousands of cycles (Supplementary Fig. 38). Compared to S-PDMS-G, P1-Si, and P2-Si, the COF of P1-Si still dropped by over 60%. We infer that the old ball with products still works. To verify that, we changed the new ball after the mixed liquid on the surface was wiped off by the dust-free fabric and performed a repeated friction test. The friction results show that a running-in period of thousands of cycles is necessary under this condition (Supplementary Fig. 39). Overall, all these indicate that this porous surface has the potential for long-term service.

2.4 Supplementary mechanical performance and porous structures

We studied the mechanical properties of HN-PDMS@BA as shown in Supplementary Fig. 41A. Results of tensile fracture experiments with different proportions of HN-PDMS@BA reveal that the tensile limit of the heterogeneous-network elastomer is generally higher than that of the single network S-PDMS, following the introduction of D-PDMS@BA into the S-PDMS network. HN-PDMS@BA/10% exhibited the maximum breaking elongation (125% higher than PDMS). The breaking strength and toughness of HN-PDMS@BA/11% were increased by 36% and 140%, respectively (Supplementary Fig. 51A). The tensile modulus of HN-PDMS@BA could be adjusted from 0.8 MPa to 1.5 MPa (Supplementary Fig. 41B). Supplementary Figs. 51B-H shows all samples exhibit very low energy dissipation. Moreover, as the number of stretching cycles increased, the energy dissipation became smaller (Supplementary Fig. 51I). As the proportion of D-PDMS@BA decreased, the energy dissipation coefficient also decreased (Supplementary Fig. 52). The lowest energy dissipation coefficient was 0.73% among all HN-PDMS@BAs. Furthermore, under step cyclic stretching, the energy dissipation of HN-PDMS@BAs decreases. Meanwhile, the tensile modulus of S-PDMS decreased slightly and that of HN-PDMS@BAs slightly increased on the contrary (Supplementary Fig. 53). This was because a part chain of PDMS breaks, resulting in a

decrease in the tensile modulus. The molecular chain orientation of HN-PDMS@BA tended to be more inclined to the tensile direction due to the existence of the D-PDMS@BA network, which improved the tensile modulus. Besides, the tensile modulus can be adjusted from 1.5 MPa to 1.9 MPa with the change of temperature (Supplementary Fig. 54). After cyclic compression experiments, we found that the dissipation energy of HNP-PDMS was lower than that of S-PDMS (18.5 kJ/m^3) as shown in Supplementary Fig. 55. Especially for HNP-PDMS@bPdBA, it has the lowest dissipation energy of ~3.1 kJ/m^3. SPDMS and HNP-PDMS@bPdBA showed the highest and lowest compressive strength, respectively (Supplementary Fig. 56). The low compressive strength and high tensile strength of HNP-PDMS@bPdBA result in a difference of ~1.7 MPa between the compressive and tensile modulus. HNP-PDMS@PdBA and HNP-PDMS@bPdBA elastomers both presented porous surfaces, and the size of their pores was larger than that of HNP-PDMS@BA (Supplementary Fig. 57).

2.5 Supplementary Evidence of Generically Improved Mechanical Properties

As shown in Supplementary Fig. 43B, HNP-PDMS@bPdBA cured in -0.1MPa/25 °C shows lowest tensile modulus. Low entanglement and crosslinking density generally result in low modulus and low elongation[8,9]. To investigate the cause of the high stretch, we performed dynamic mechanical analysis tests. Loss factor ($tan\delta$), representing the degree of viscous deformation, can be calculated according to the following formula:

$$tan\delta = M''/M' \tag{21}$$

where M' and M'' are storage modulus and loss modulus. Storage modulus and loss modulus represent viscosity and elasticity. The $tan\delta$ of HNP-PDMS@bPdBA cured in -0.1MPa/25°C is much larger than that of HNP-PDMS@bPdBA cured in -0.1MPa/125°C and -0.0MPa/25°C (Supplementary Fig. 44). Therefore, we believe that viscoelastic effect leads to the high stretch of HNP-PDMS@bPdBA cured in -0.1MPa/25°C. The stretch percentage could be improved to 1250%, which is attributed to the increase in the degree of viscous deformation of HNP-PDMS@bPdBA.

3. Supplementary Figures

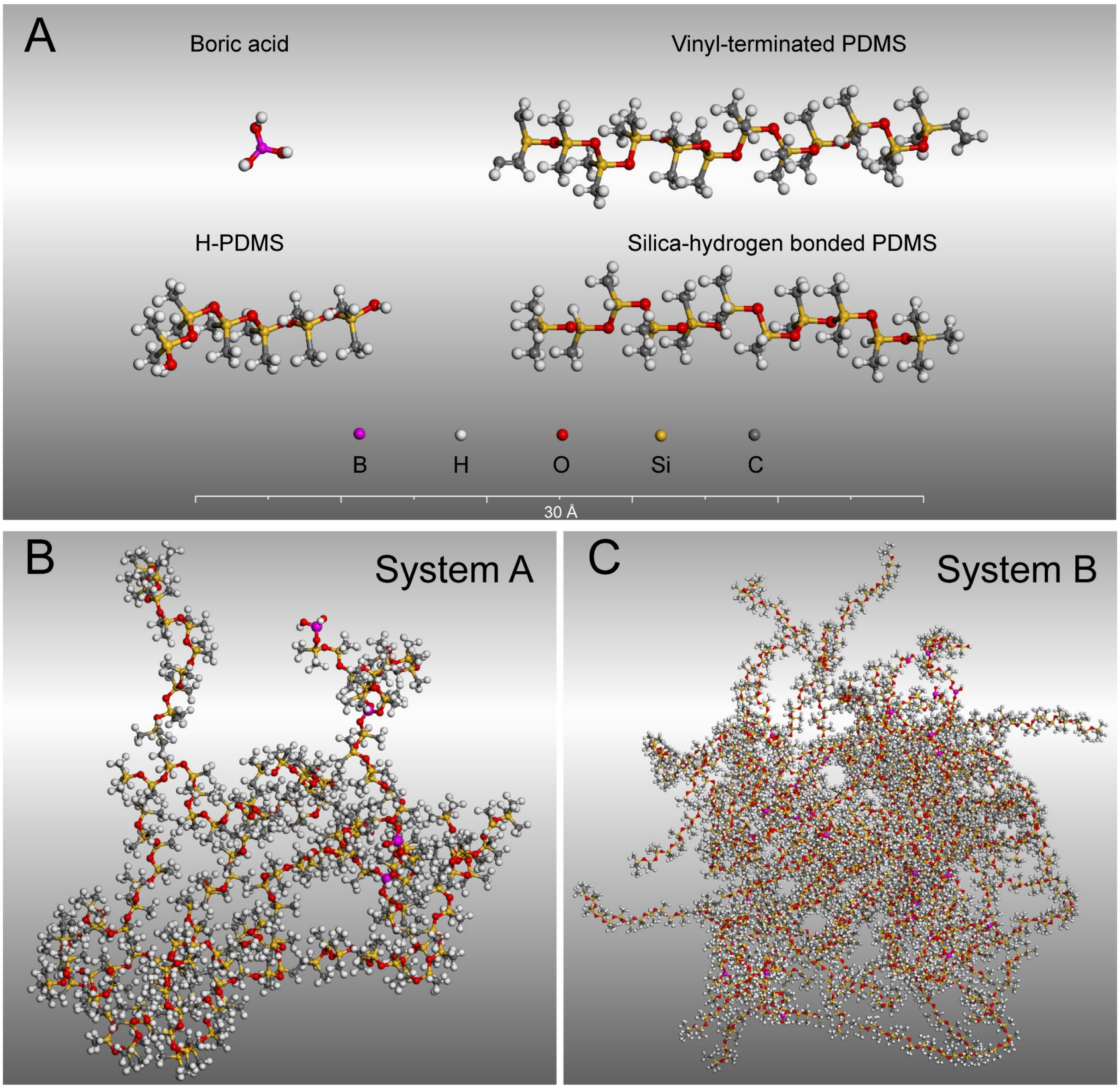

Supplementary Fig. 1. The model of MD simulation. (A) Model of a single molecule (B) D-PDMS@BA chains with 6 HPDMS and 4 boric acid molecules, S-PDMS chain with 8 vinyl-terminated PDMS and 4 silica-hydrogen bonded PDMS molecules in the system A. (C) 9 D-PDMS@BA chains and 9 S-PDMS chains in the system B.

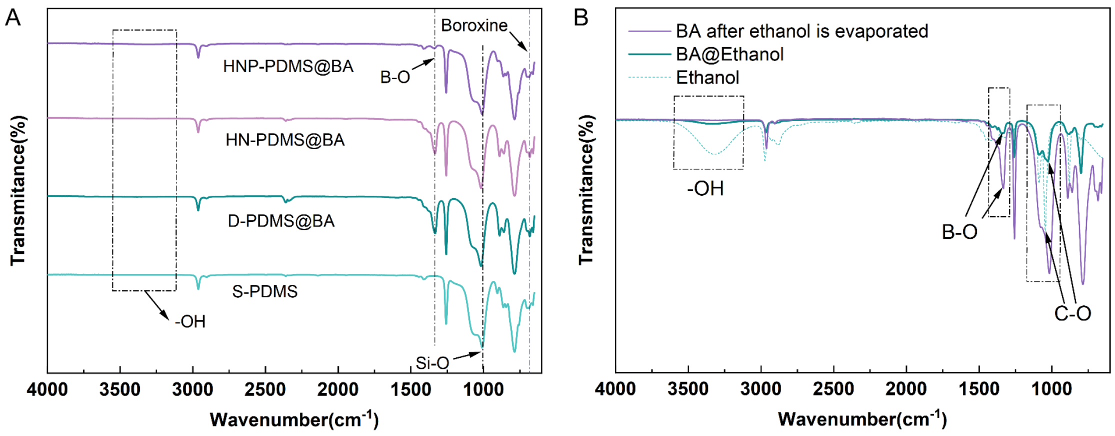

Supplementary Fig. 2. (A) Fourier transform infrared spectrum of S-PDMS, D-PDMS@BA, HN-PDMS@BA, and HNP-PDMS@BA. (B) Fourier transform infrared spectrum of ethanol, the mixture of D-PDMS@BA and ethanol, and D-PDMS@BA after ethanol evaporated.

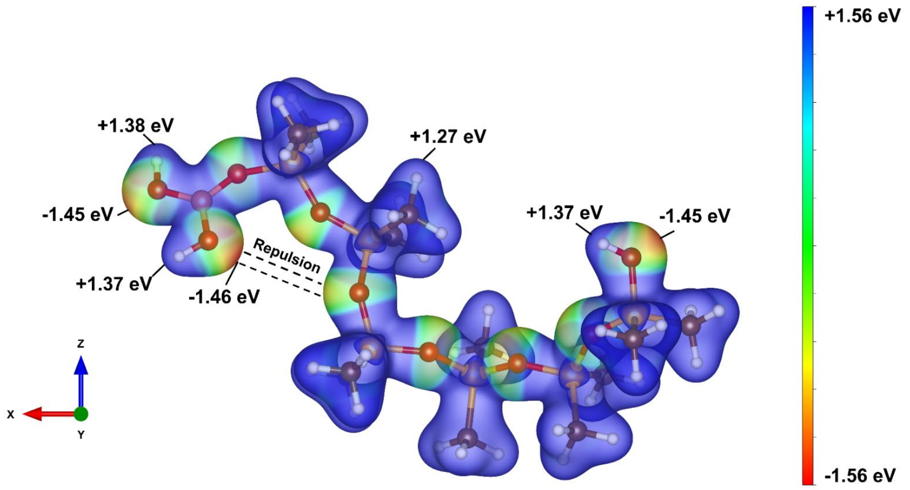

Supplementary Fig. 3. Electrostatic potential map of a repeating unit in D-PDMS@BA

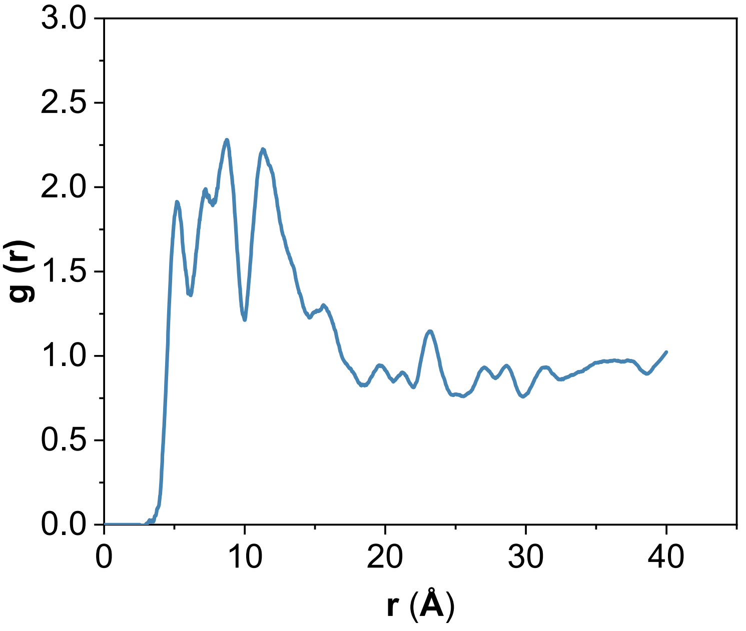

Supplementary Fig. 4. The radial distribution function of B atoms in system B.

$$3ROH + (R'O)_3B \rightleftharpoons (RO)_3B + 3R'OH$$

Supplementary Fig. 5. Reaction of borate esters with alcohols.

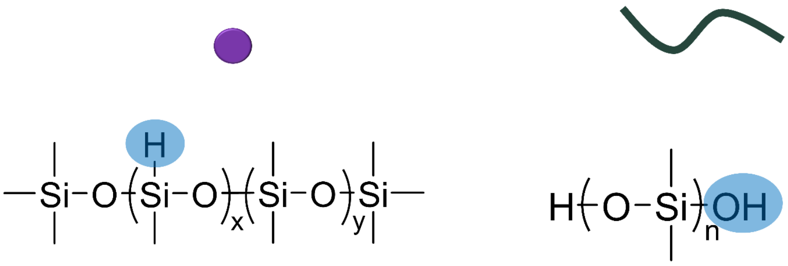

Supplementary Fig. 6. Molecular structures and reaction sites of SHPDMS and HPDMS.

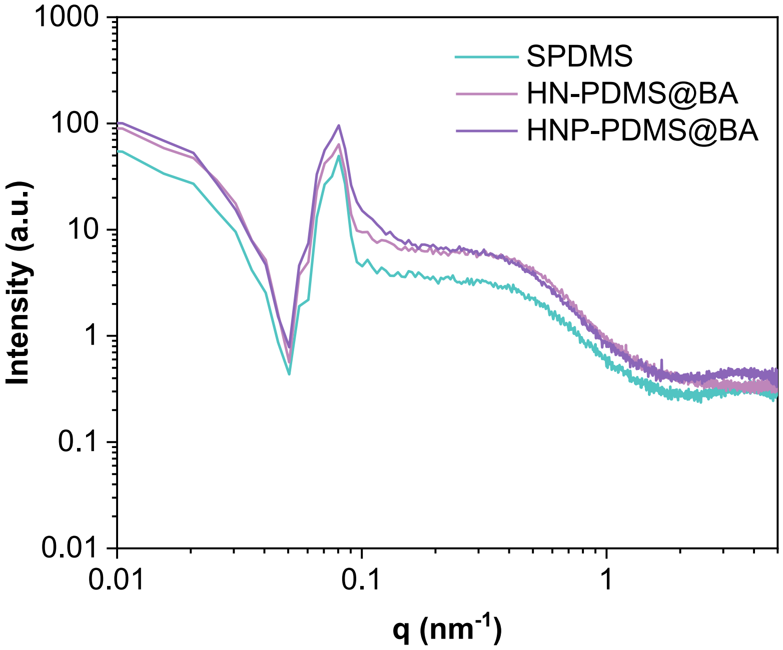

Supplementary Fig. 7. 1D SAXS scattering intensity curves of different samples. The periodicity of the phase-separated domains was calculated to be 78.5 nm ($D = 2\pi/ q^*$, q^* represents the scattering vector value corresponding to the peak).

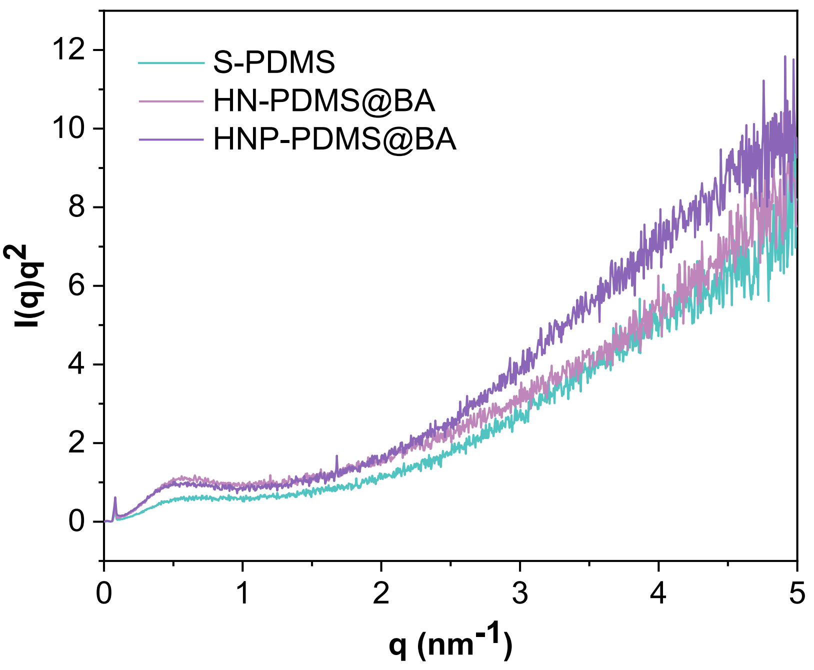

Supplementary Fig. 8. Kratky method was performed to estimate the flexibility of the molecules.

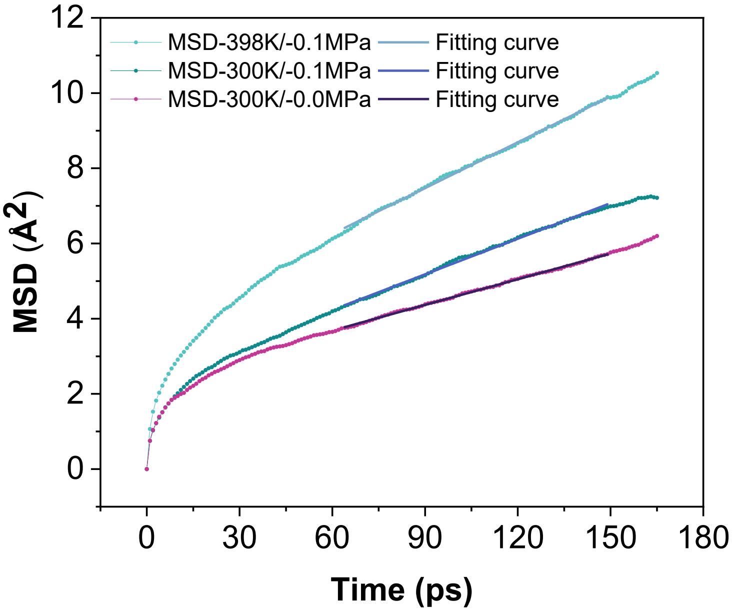

Supplementary Fig. 9. MSD of B atoms at 300 K or 398 K and -0.1 MPa or -0 MPa in the system of B.

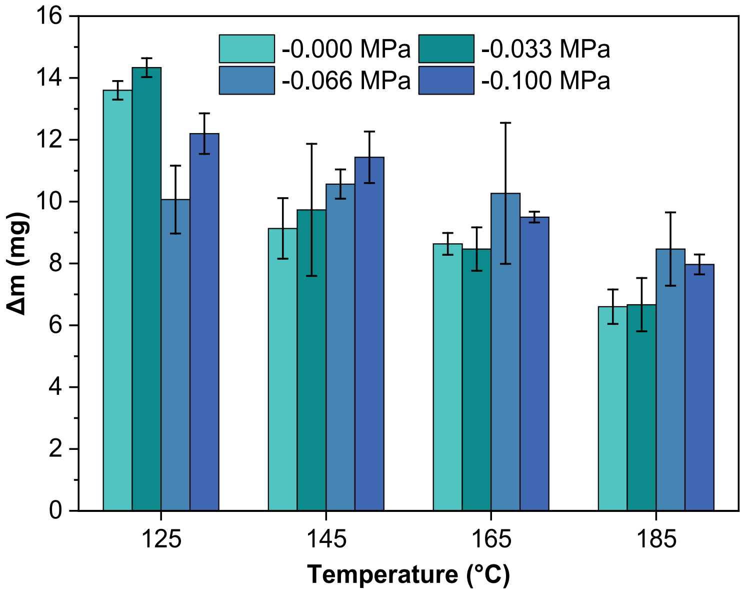

Supplementary Fig. 10. After HN-PDMS@BA were cured at different temperatures and pressures, the weight of HN-PDMS@BA changed (Δ m) when their unstable structures were removed. The error bars show the standard error of the mean, computed from at least three samples.

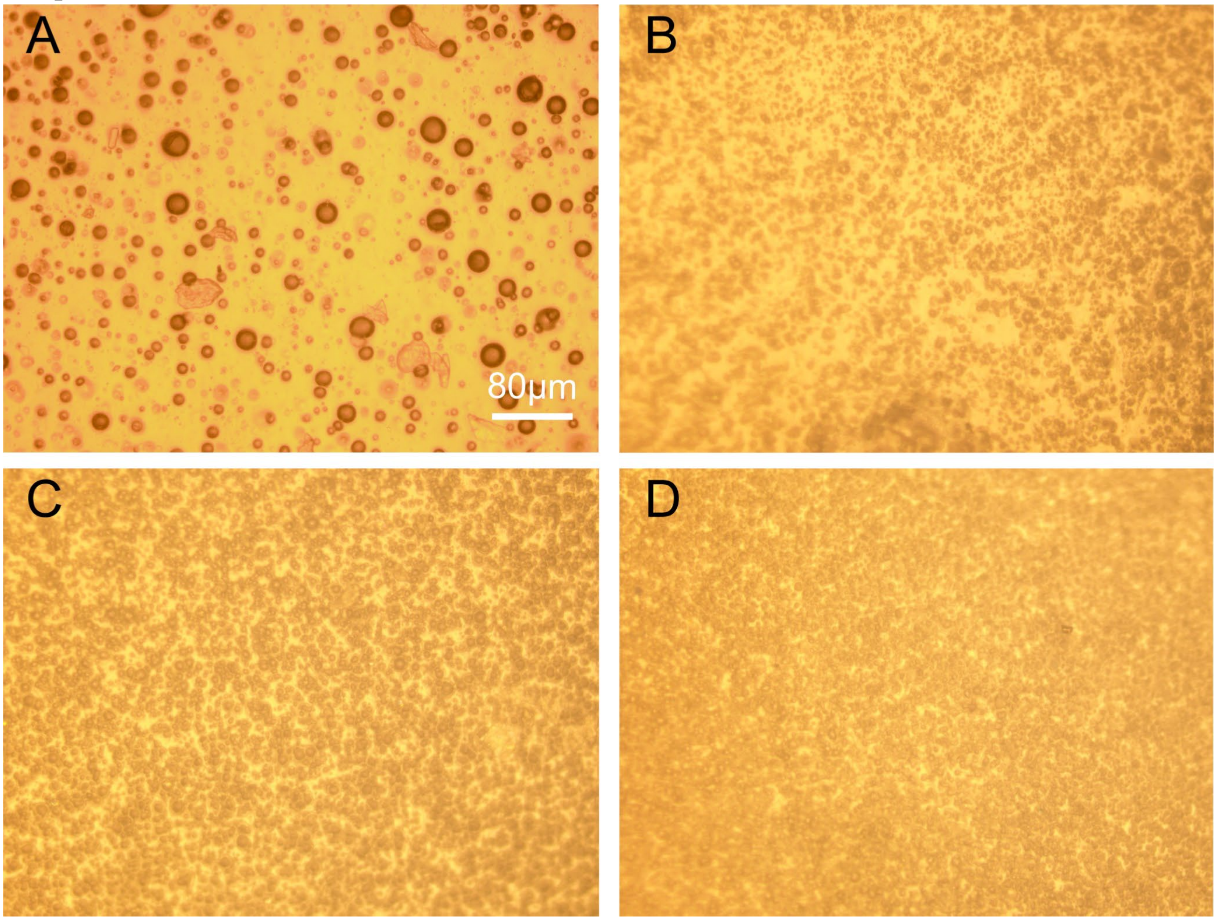

Supplementary Fig. 11. The 2D optical microscope surface of HNP-PDMS@BA cured at 125 °C under (A) -0.1 MPa, (B) -0.66 MPa, (C) -0.33 MPa, and (D) -0 MPa respectively. Scale bars are the same for all figures.

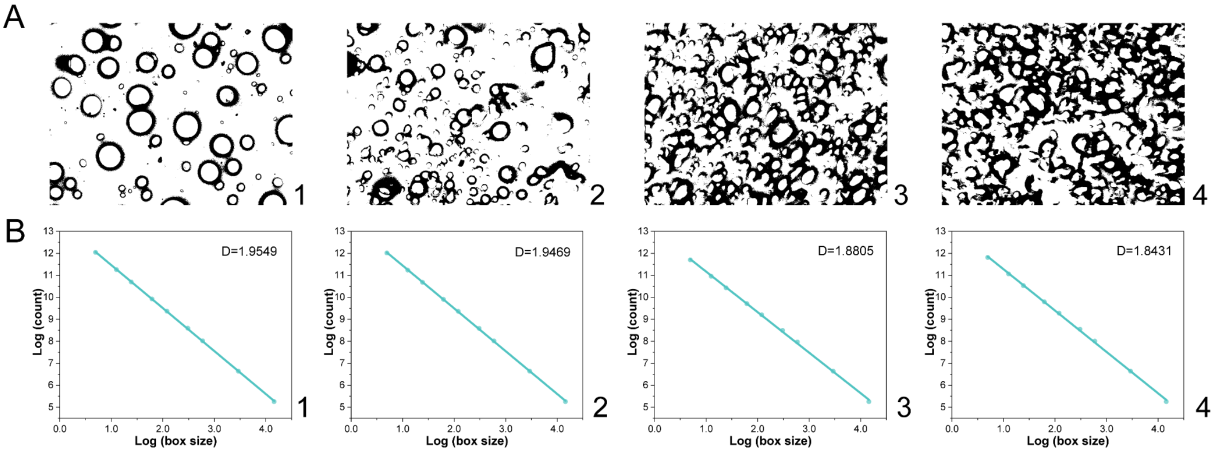

Supplementary Fig. 12. (A) The feature extraction and (B) fractal dimension diagram of pore contour of HNP-PDMS@BA surface cured under -0.1 MPa at 125 °C, 145 °C, 165 °C, and 185 °C respectively.

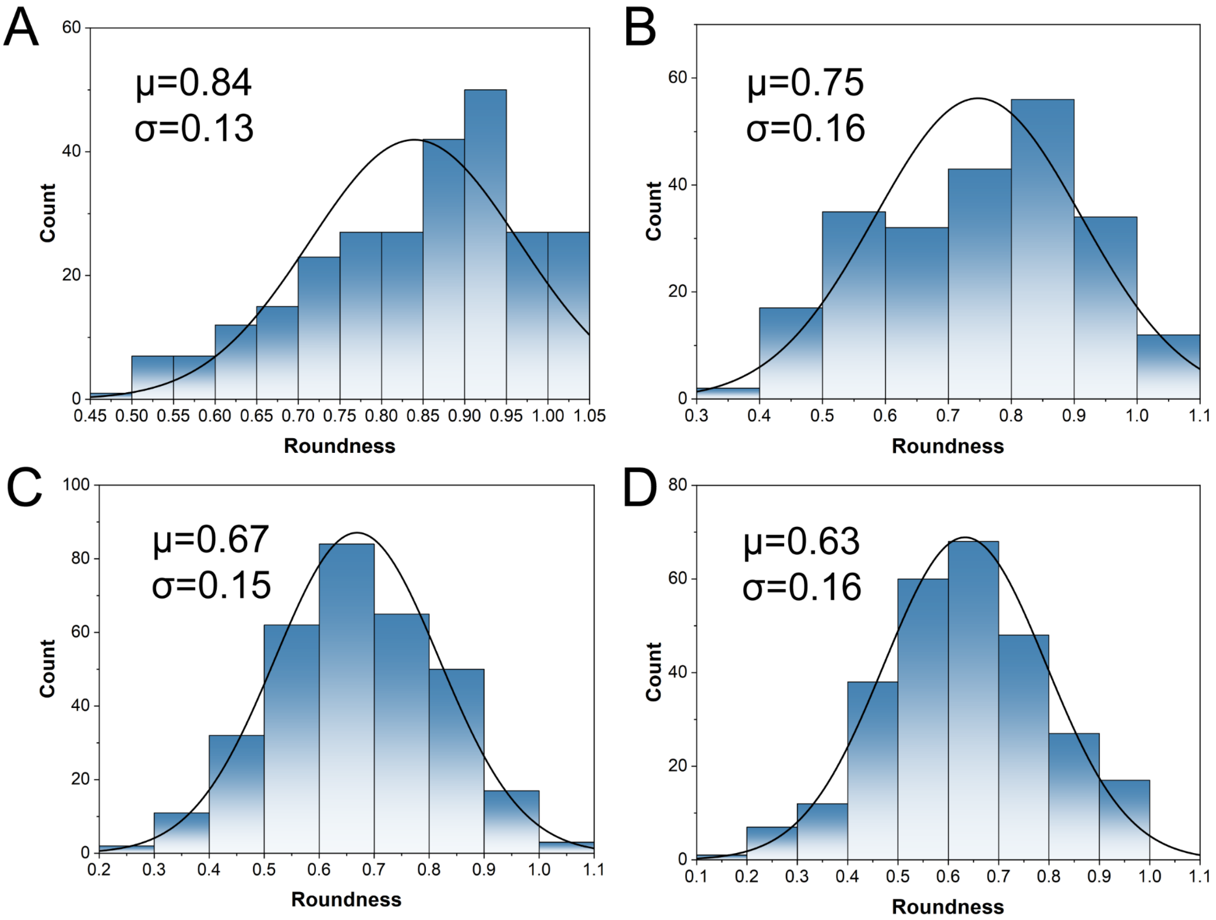

Supplementary Fig. 13. Pores' roundness of HNP-PDMS@BA surface cured under -0.1 MPa at 125 °C, 145 °C, 165 °C, and 185 °C respectively.

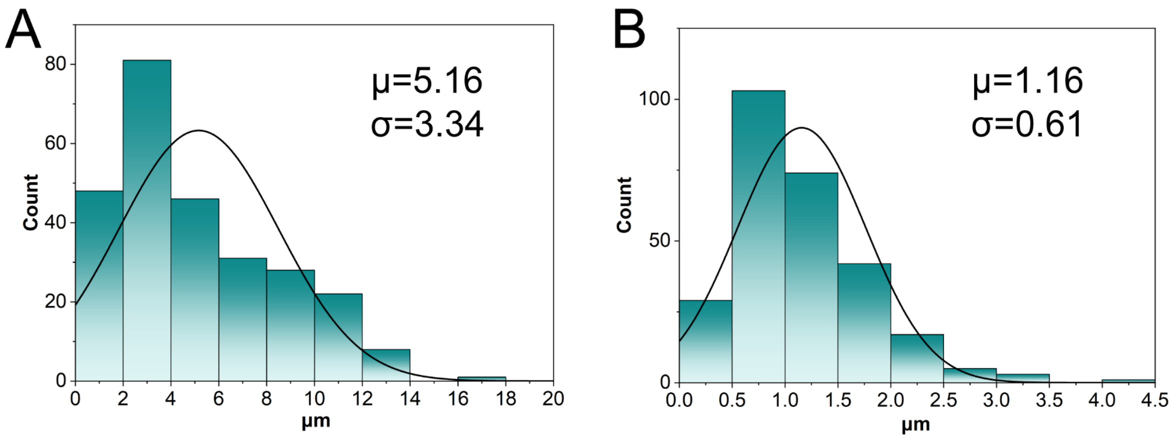

Supplementary Fig. 14. Pores' feret diameter of HNP-PDMS@BA surface cured under (A) -0.1 MPa and (B) -0.0 MPa at 125 ℃.

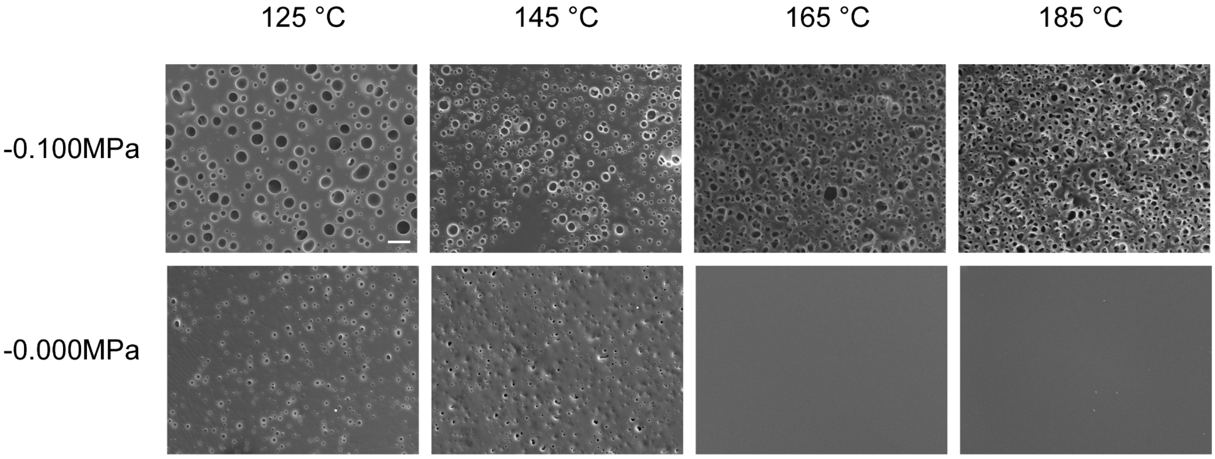

Supplementary Fig. 15. The surfaces' SEM images of HNP-PDMS@BA cured at 125 ℃ ~ 185 ℃ under -0.1 MPa or -0.0 MPa. The scale bar is 20 μm and the scale bar is the same for all figures.

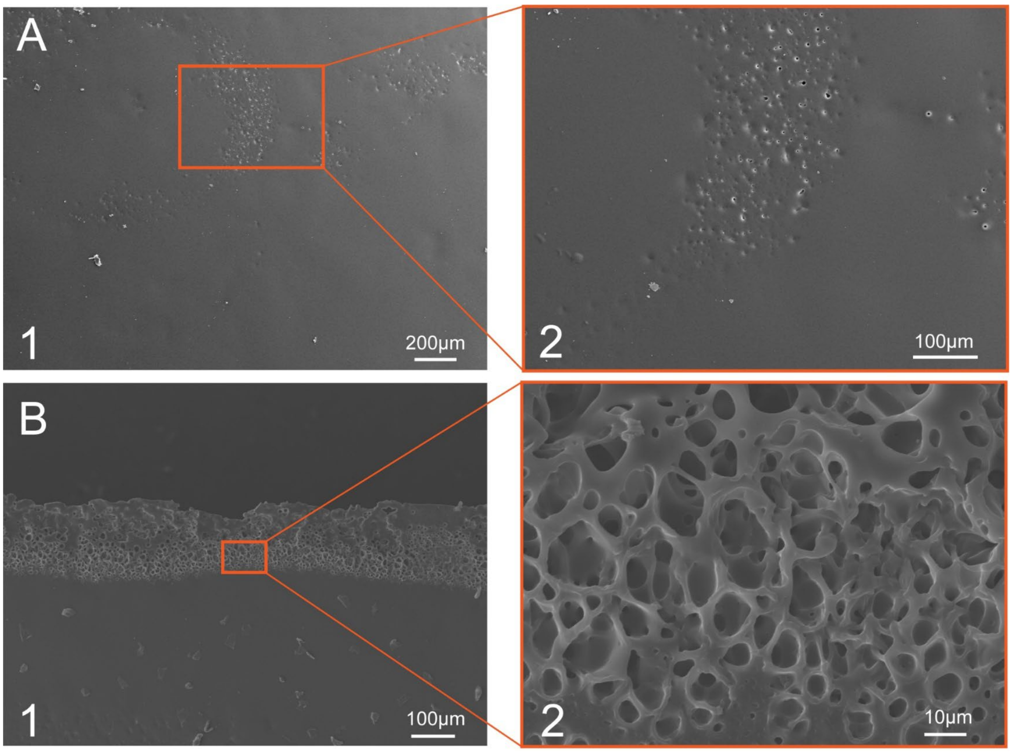

Supplementary Fig. 16. (A) The surfaces' and (B) oblique sections' SEM images of HNP-

PDMS@BA cured at 185 °C under -0.0 MPa.

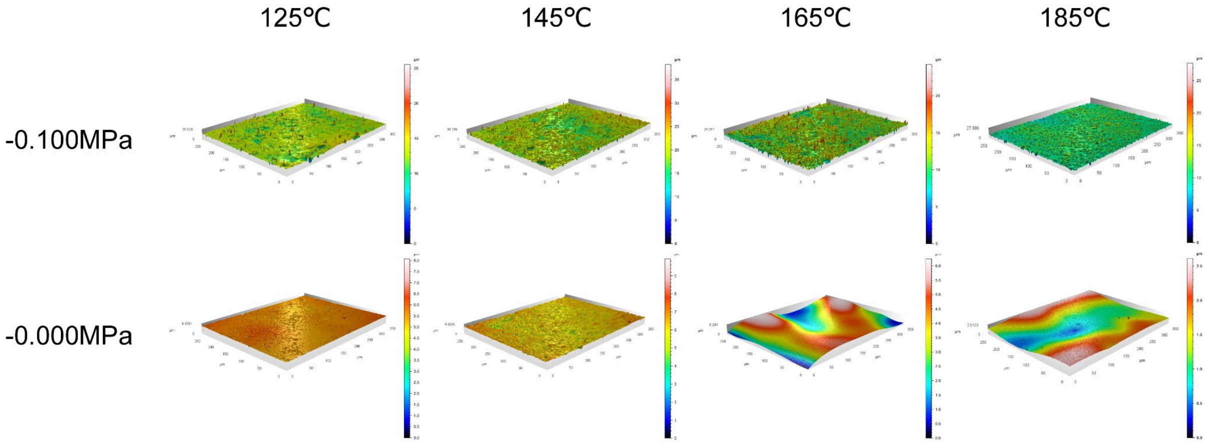

Supplementary Fig. 17. The surfaces' 3D contour images of HNP-PDMS@BA cured at 125°C ~ 185°C under -0.1MPa or -0MPa.

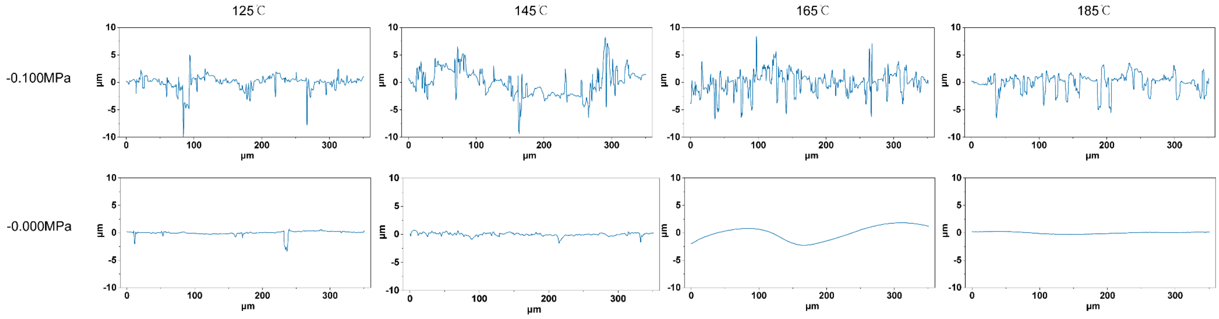

Supplementary Fig. 18. The surfaces' 3D contour images of HNP-PDMS@BA cured at 125 °C ~ 185 °C under -0.1 MPa or -0.0 MPa.

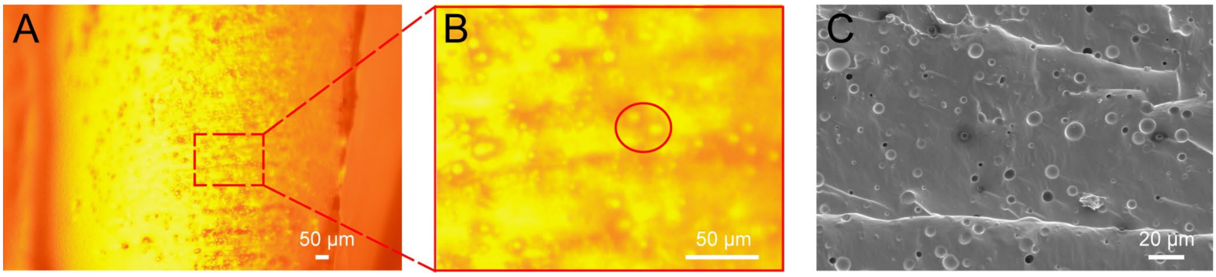

Supplementary Fig. 19. (A), (B) The optical microscope images of oiled HNP-PDMS@BA cross-section and (C) SEM image of unoiled HNP-PDMS@BA cross-section.

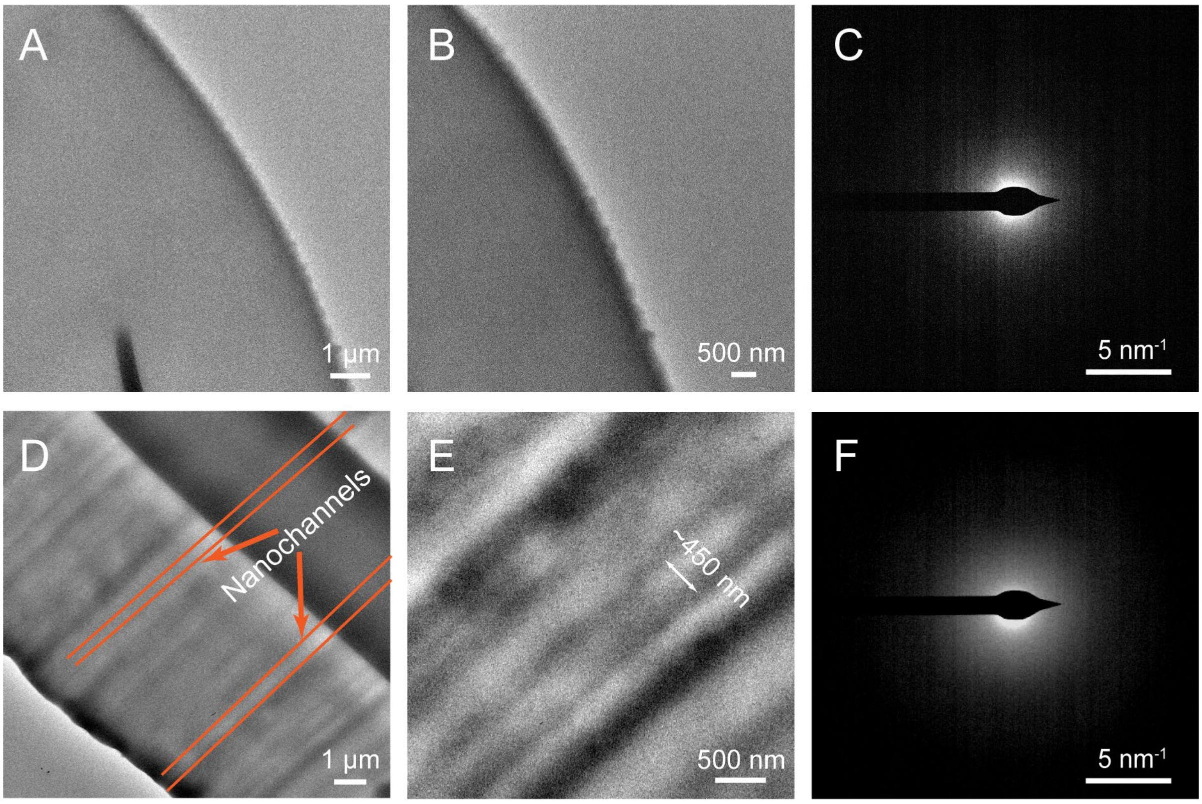

Supplementary Fig. 20. TEM images and corresponding electron diffraction of (A)-(C) S-PDMS and (D)-(F) HNP-PDMS@BA.

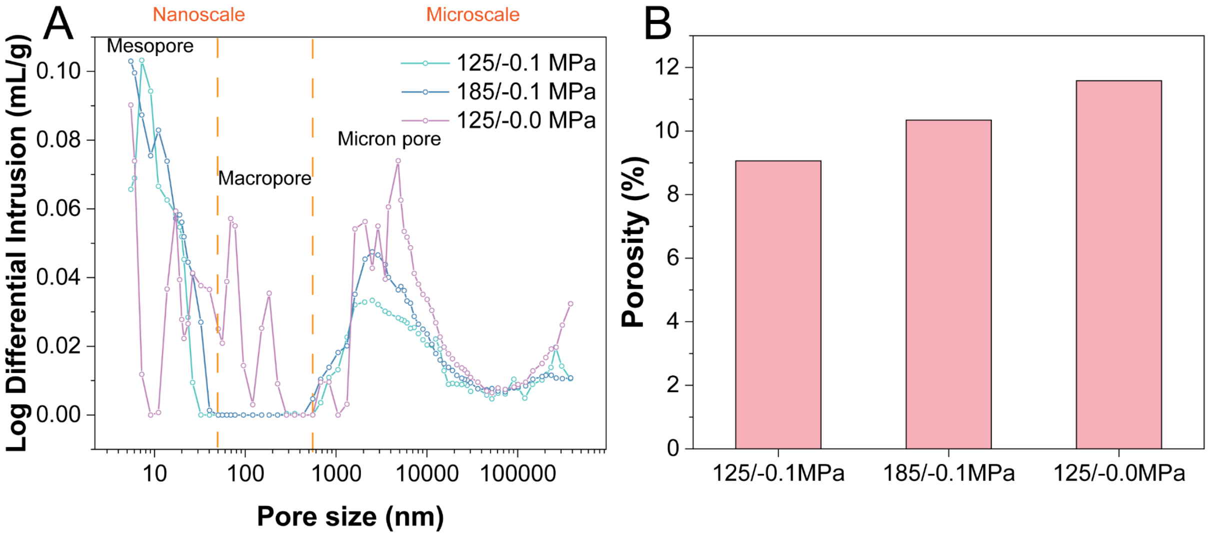

Supplementary Fig. 21. (A) The curves of pore size and log differential intrusion and (B) porosity for HNP-PDMS@BA cured at different temperatures and pressures.

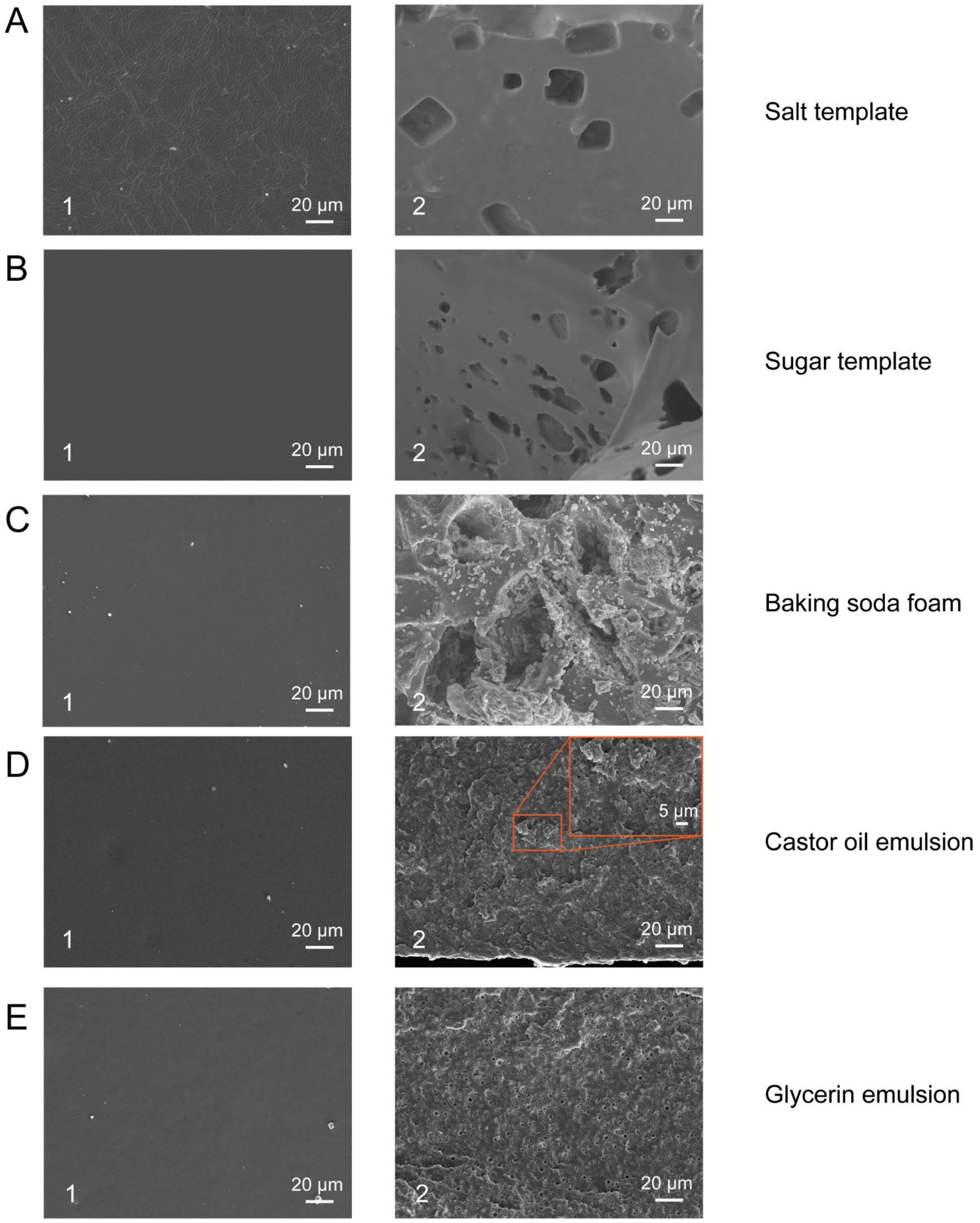

Supplementary Fig. 22. The surface and the section SEM images of porous elastomers via different methods of preparation.

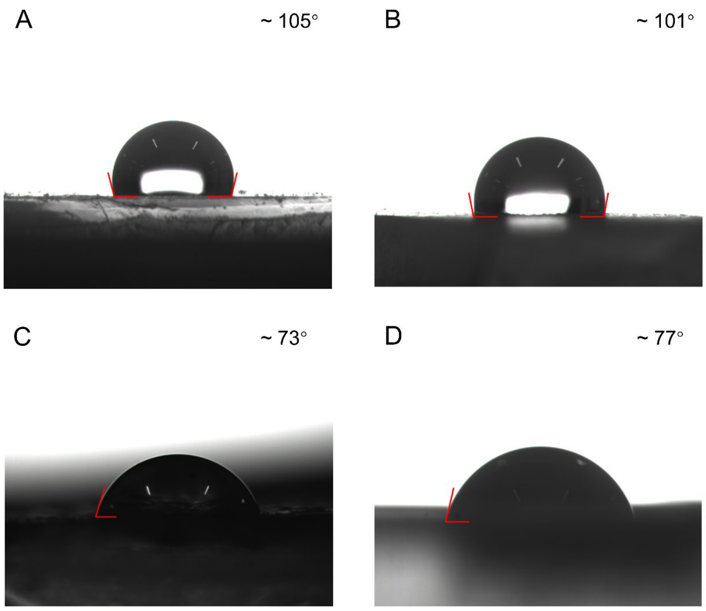

Supplementary Fig. 23. The wetting status of water droplets on the (A) S-PDMS surface, (B) P1 surface, (C) P1 surface after one friction experiment, and (D) P1 surface after three repeated friction experiments.

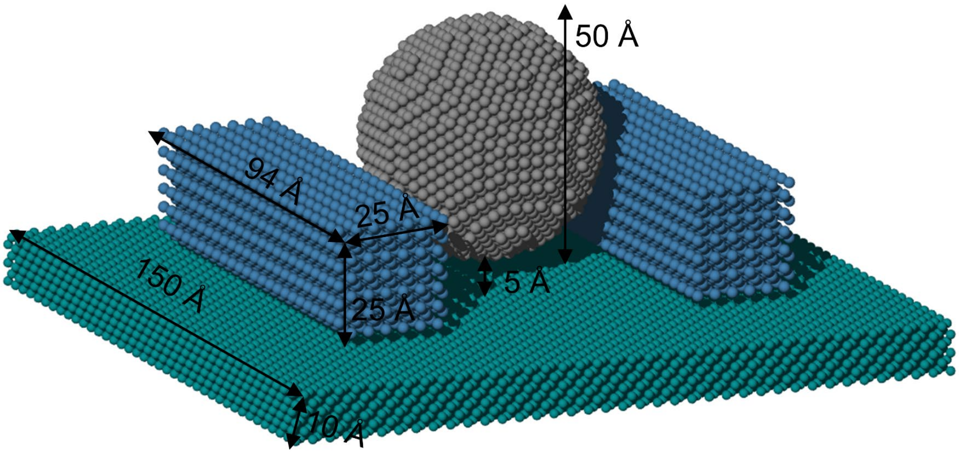

Supplementary Fig. 24. The model of water under the interaction of a steel ball with a hydrophobic or hydrophilic surface (Blue particles: water molecules; Gray particles: iron atoms; The cyan surface was used to simulate a hydrophobic or hydrophilic surface).

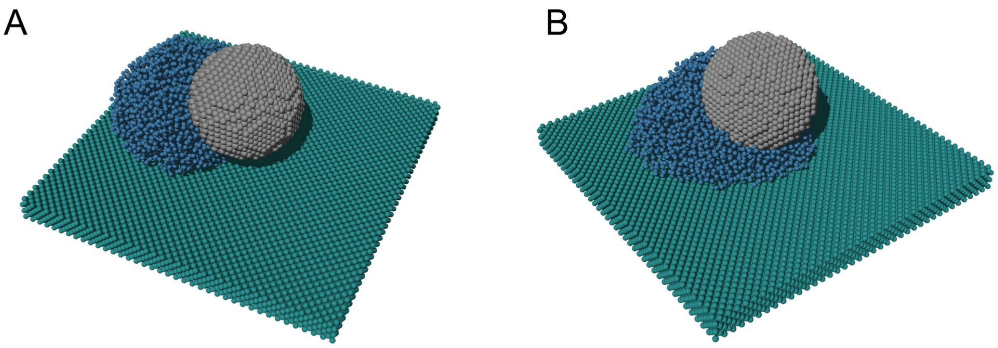

Supplementary Fig. 25. Perspective snapshot of water interacting with a steel ball and a hydrophobic or hydrophilic surface at 10 ns.

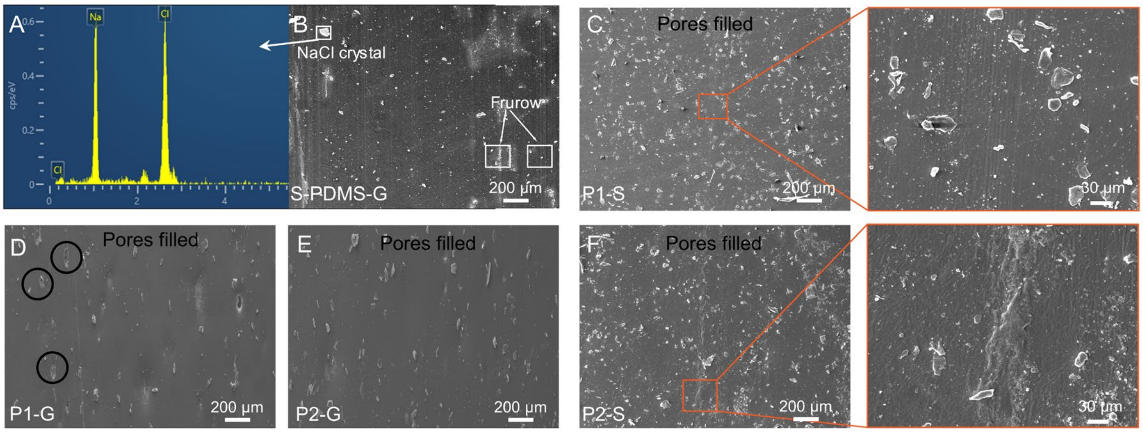

Supplementary Fig. 26. (A) Energy dispersive spectroscopy result. SEM images of (B)S-PDMS-G, (C)P1-S, (D)P1-G, (E)P2-G, and (F) P2-S

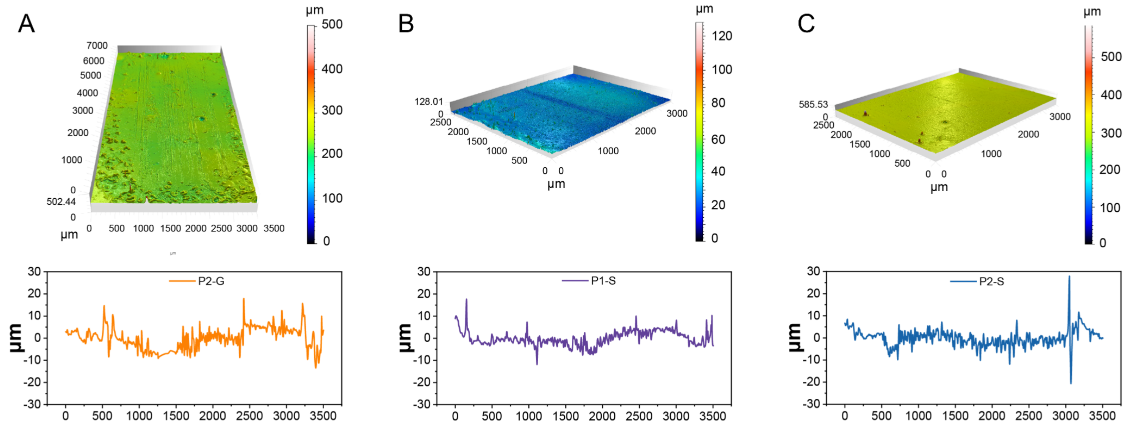

Supplementary Fig. 27. The 3D profile and 2D cross-section of (A) P2-G, (B) P1-S, and (C) P2-S after 14400 cycles of friction.

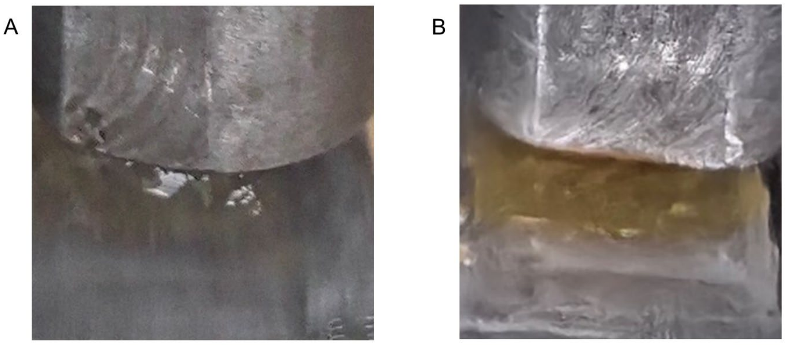

Supplementary Fig. 28. The state of salt water when (A) the beginning of friction and (B) COF decreases sharply.

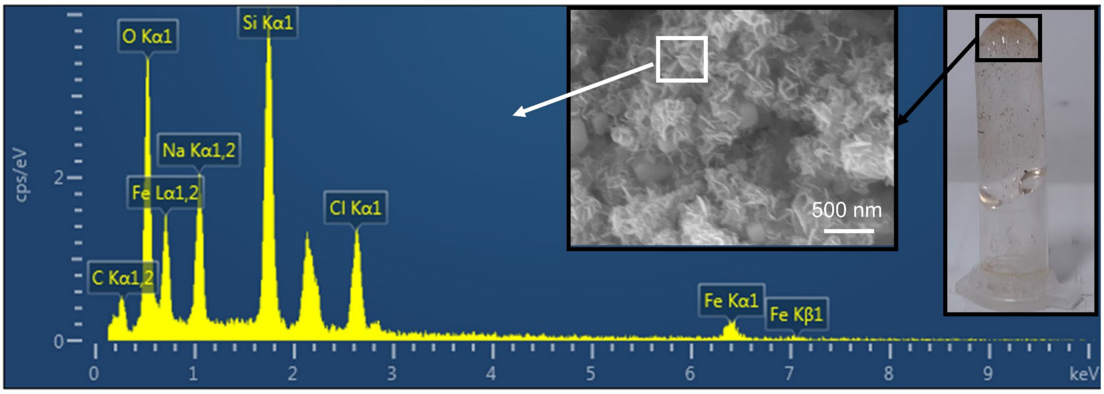

Supplementary Fig. 29. Energy dispersive spectroscopy result of wear debris. The embedded images are wear-debris SEM image and optical image (The water was adsorbed to the upper part of the centrifuge tube and did not fall down).

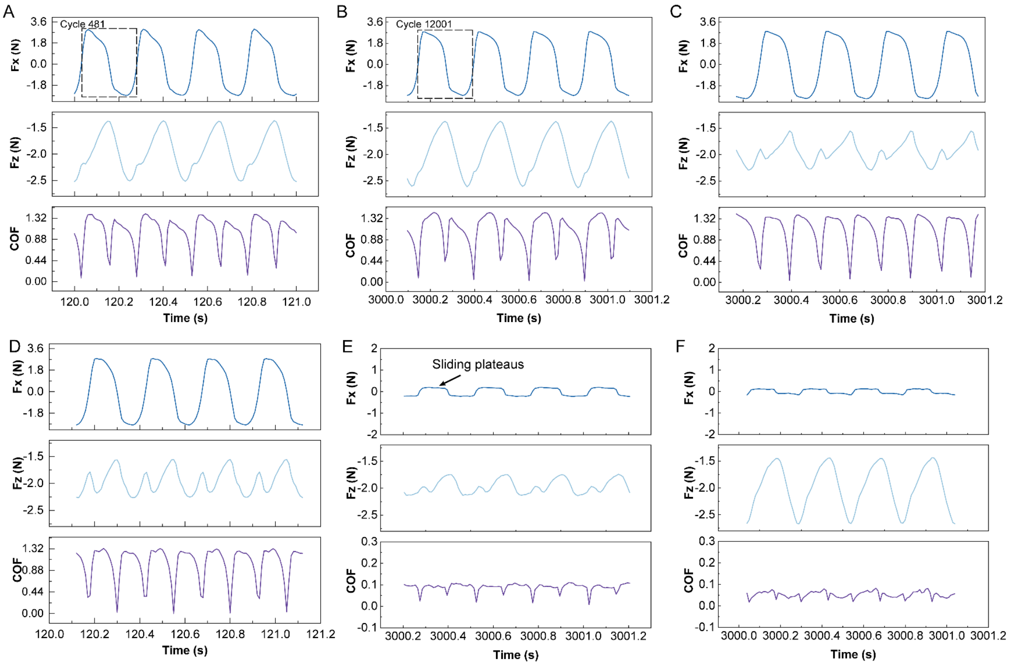

Supplementary Fig. 30. Load status and the COF of (A) S-PDMS-G and (D) P1-G and in cycles 481-484. Load status and the COF of (B) S-PDMS-G, (C) P1-Si_3N_4, (E) P1-G, and (F) P2-G in cycles 12001-12004.

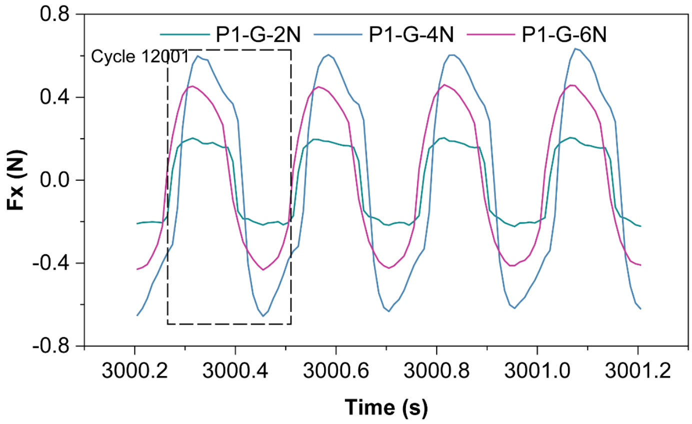

Supplementary Fig. 31. Changes in shear force (Fx) under different applied loads

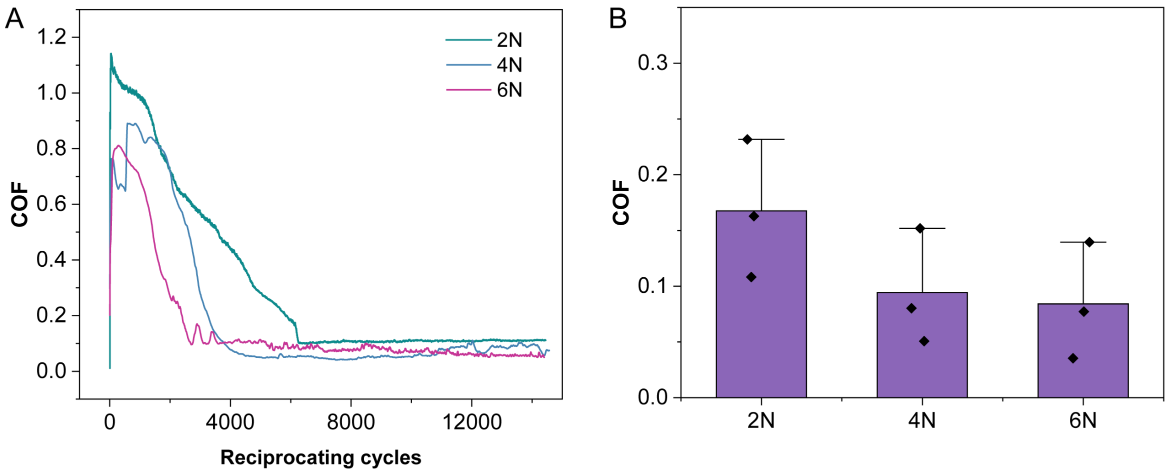

Supplementary Fig. 32. Changes in shear force (Fx) under different applied loads. The error bars show the standard error of the mean, computed from three samples.

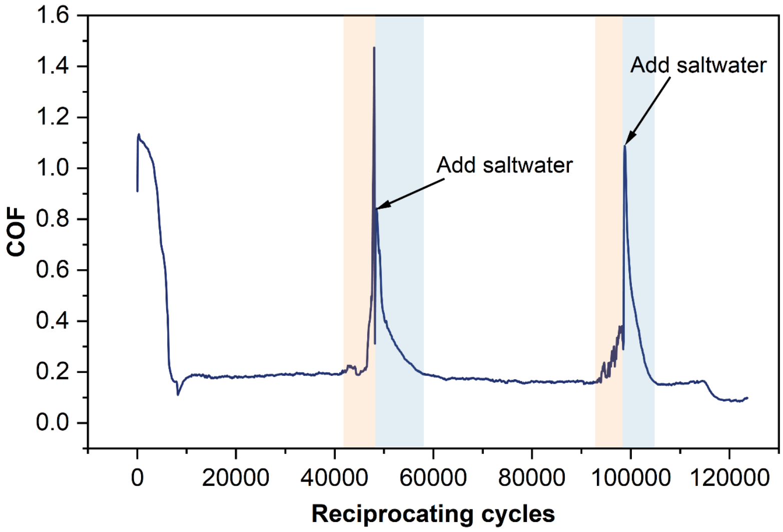

Supplementary Fig. 33. The COF–cycle curve of P1-G after 122400 reciprocating cycles (Yellow area: Lubrication deteriorated as the water evaporated; Blue area: Lubrication improved after adding 0.1 mL of salt water).

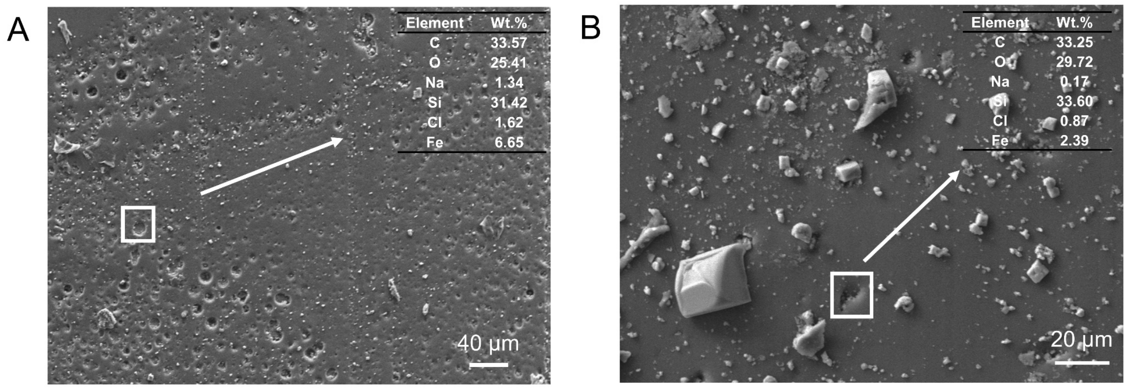

Supplementary Fig. 34. Results of SEM and EDS elemental analysis after (A) 122400 reciprocating cycles and (B) three consecutive friction tests.

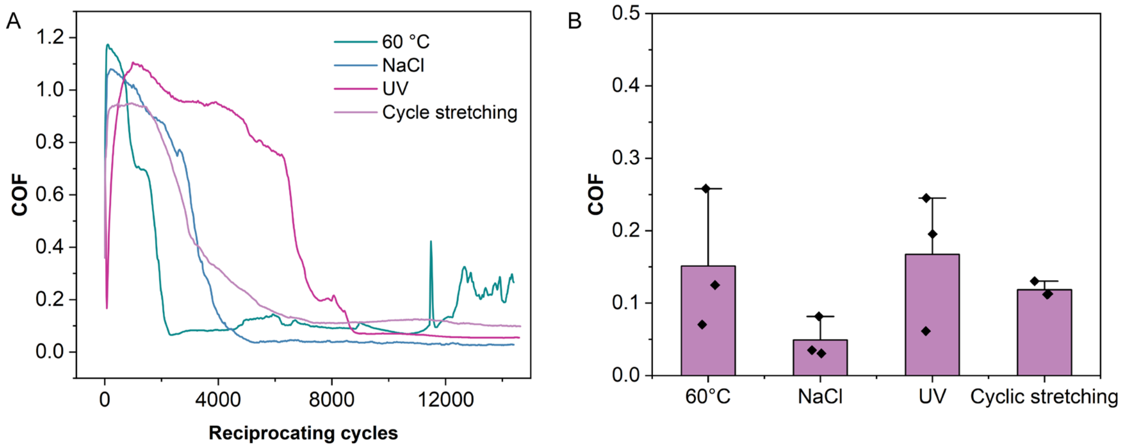

Supplementary Fig. 35. (A) The COF–cycle curve and (B) average COF of P1-G after aging or degradation treatment. The error bars show the standard error of the mean, computed from three samples.

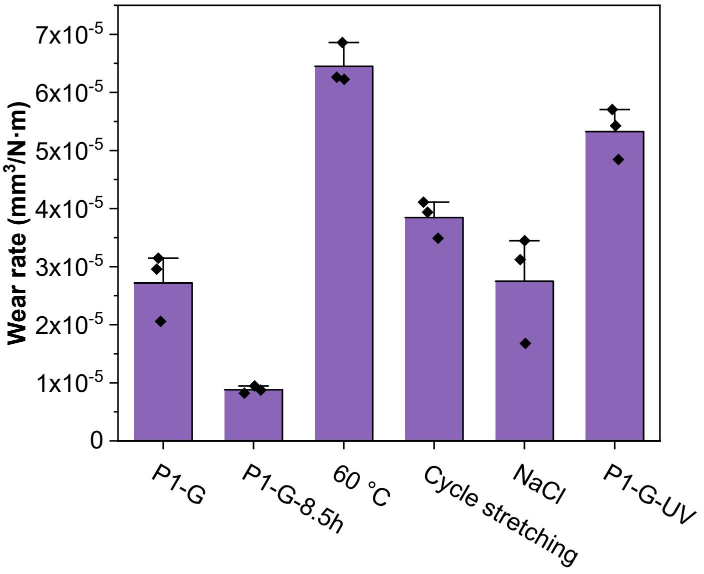

Supplementary Fig. 36. P1-G-8.5h represents the sample after 122400 cycles of friction. The error bars show the standard error of the mean, computed from three samples.

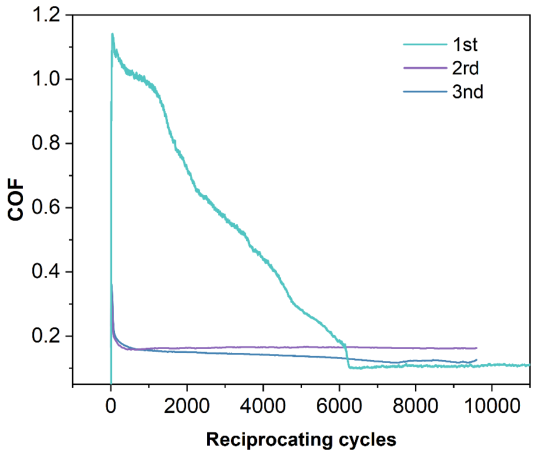

Supplementary Fig. 37. After a 12000-cycle friction test, the friction test was repeated without any treatment.

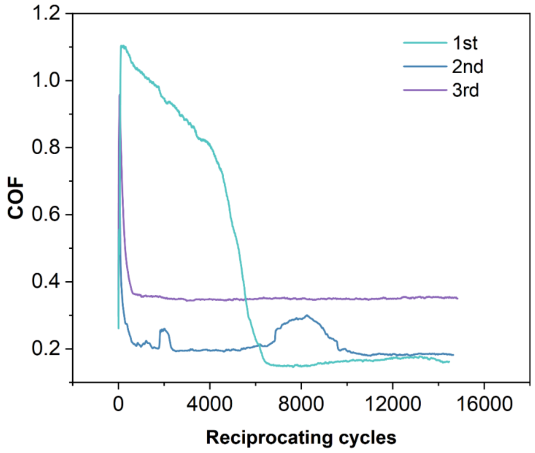

Supplementary Fig. 38. After the first friction test, wipe off the surface mixture without changing the ball: repeat the test.

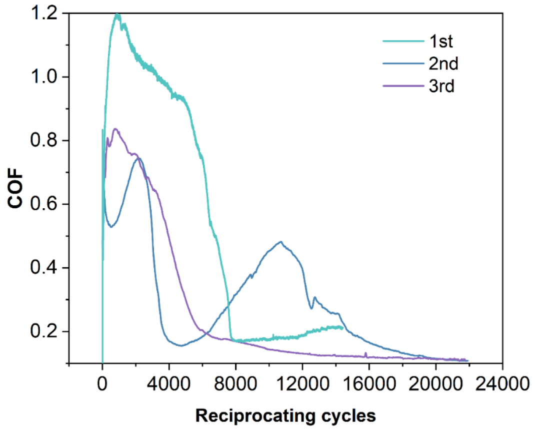

Supplementary Fig. 39. After the first friction test, wipe off the surface mixture and replace the ball with a new one: repeat the test.

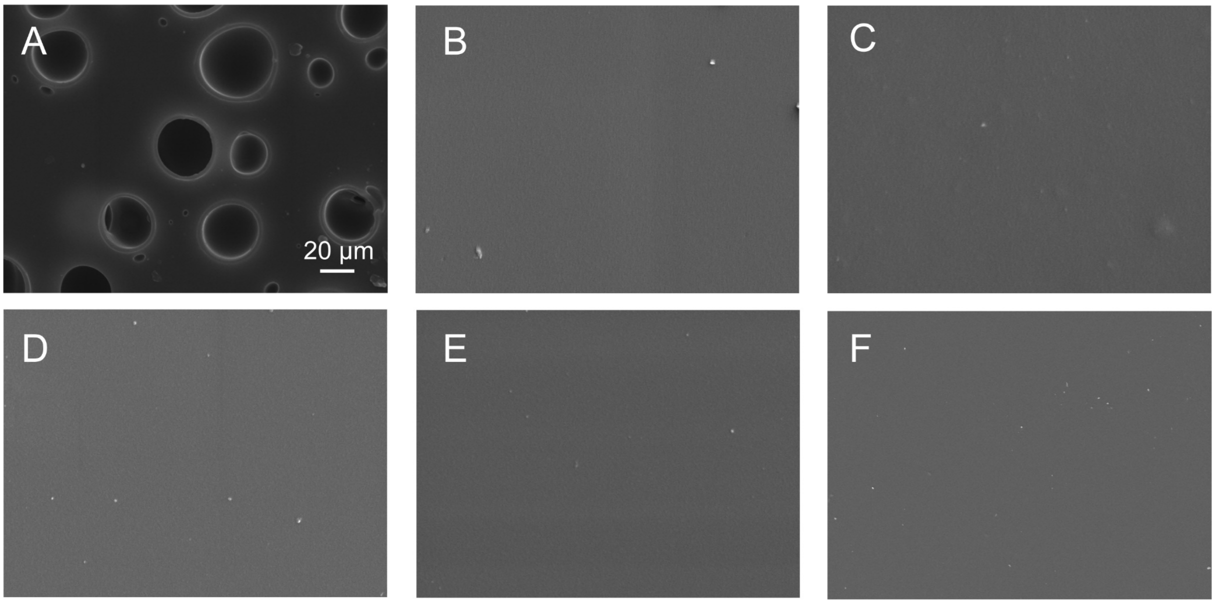

Supplementary Fig. 40. SEM images of HNP-PDMS@BA surface when the mass fraction of D-PDMS@BA in S-PDMS is respectively (A) 20%, (B) 16%, (C) 13%, (D) 11%, (E) 10%, and (F) 8%. Scale bars are the same for all figures.

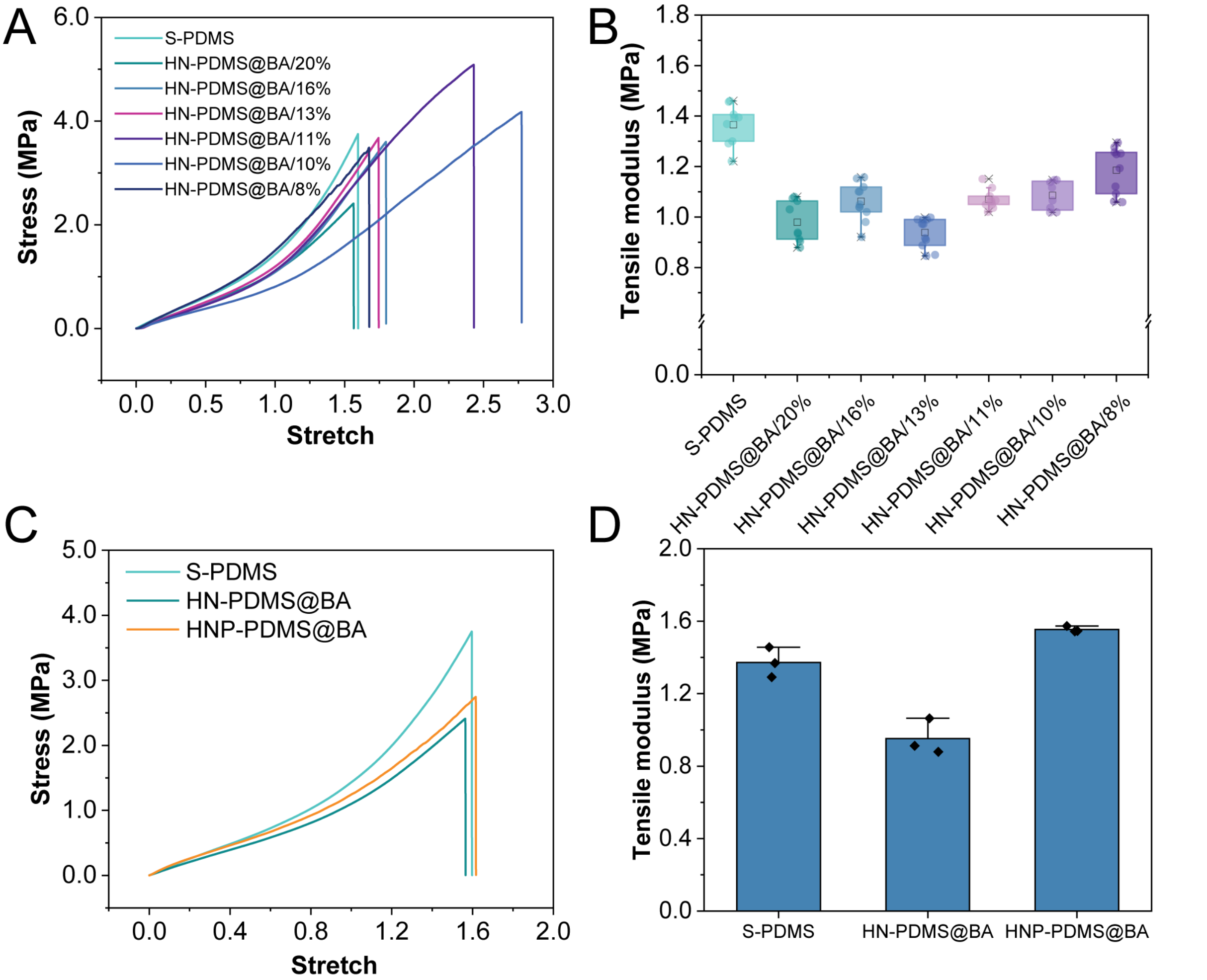

Supplementary Fig. 41. (A) Stress-stretch curves and (B) tensile modulus of different PDMS elastomers. (C) Stress-stretch curves and (D) tensile modulus of S-PDMS, HN-PDMS@BA, and HNP-PDMS@BA. The error bars show the standard error of the mean, computed from three samples.

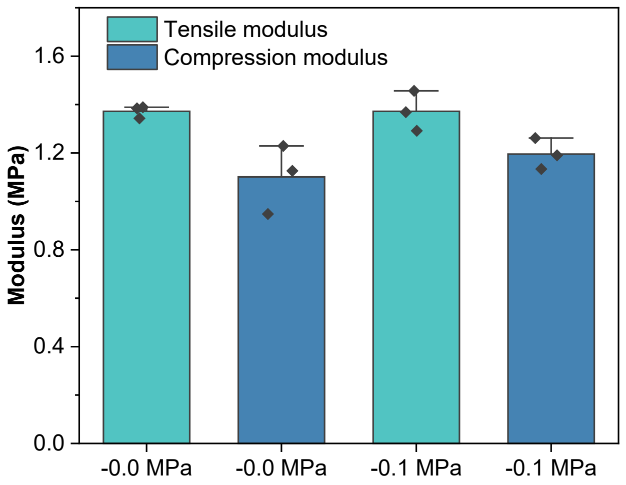

Supplementary Fig. 42. Tensile modulus and compression modulus of S-PDMS cured at 125 °C under different pressures. The error bars show the standard error of the mean, computed from three samples.

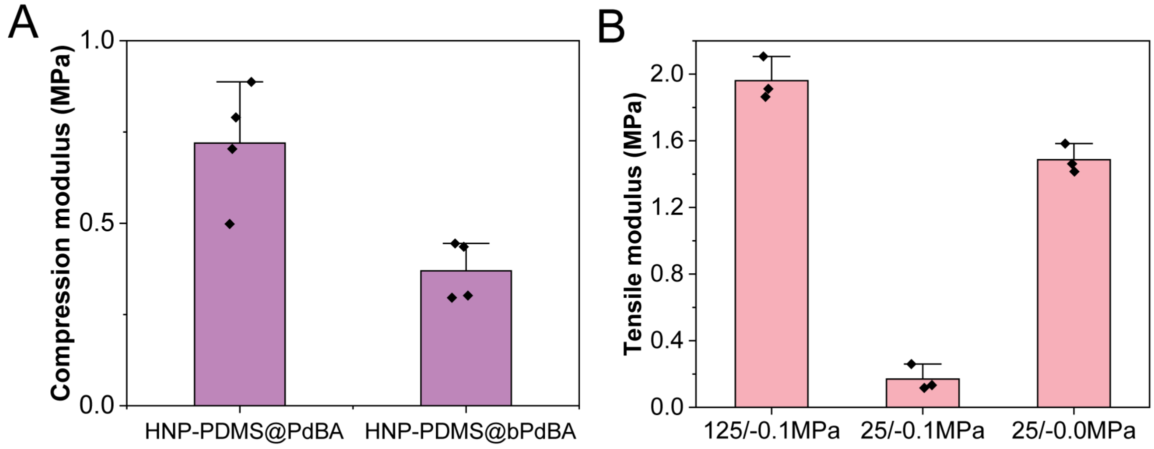

Supplementary Fig. 43. (A) Compression modulus of HNP-PDMS@PdBA and HNP-PDMS@bPdBA elastomers. (B) Tensile modulus of HNP-PDMS@bPdBA elastomers under varying pressure and temperature conditions. The error bars show the standard error of the mean, computed from three samples.

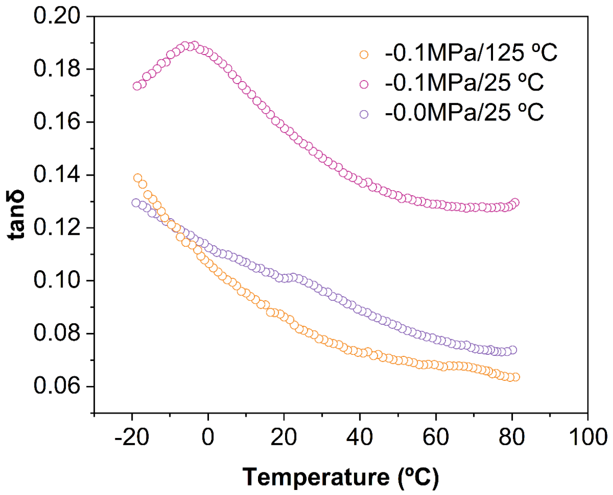

Supplementary Fig. 44. Changes in the $tan\delta$ of HNP-PDMS@bPdBA cured under different conditions with temperature.

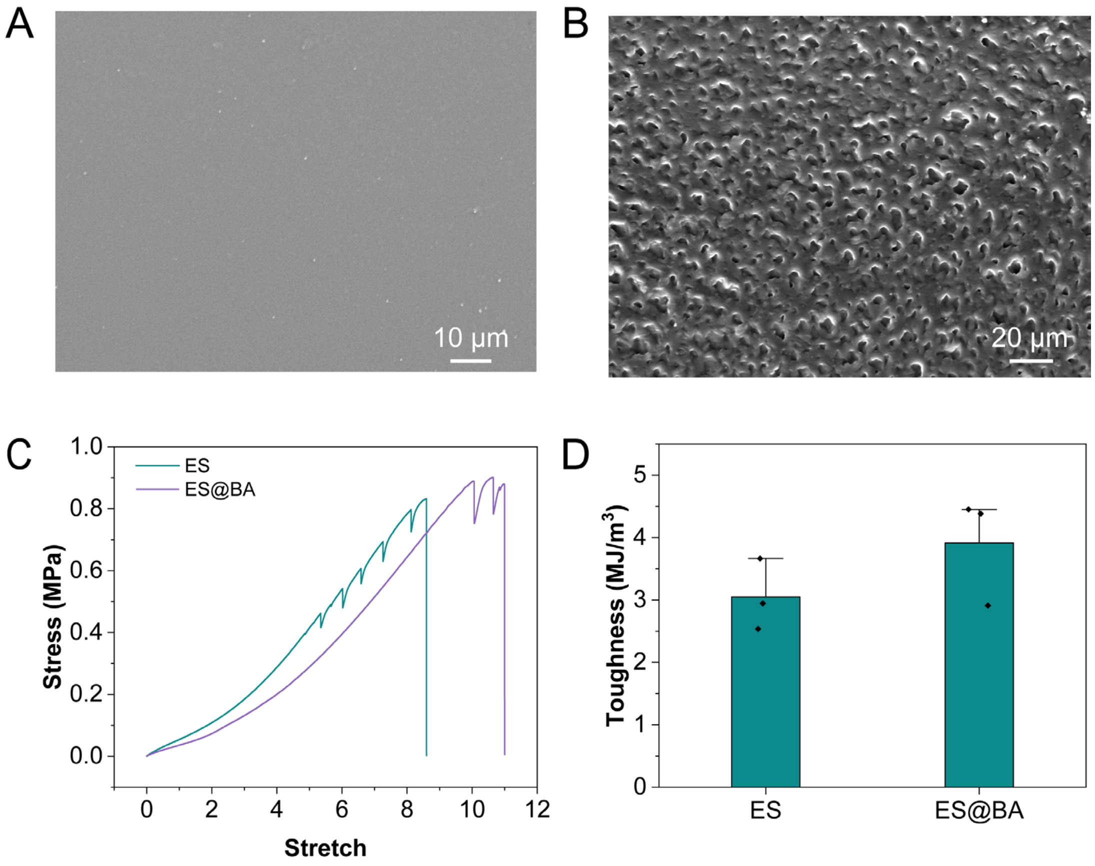

Supplementary Fig. 45. The surface SEM images of (A) ES and (B) ES@BA. (C) Stress-stretch curves. (D) Toughness comparison. The error bars show the standard error of the mean, computed from three samples.

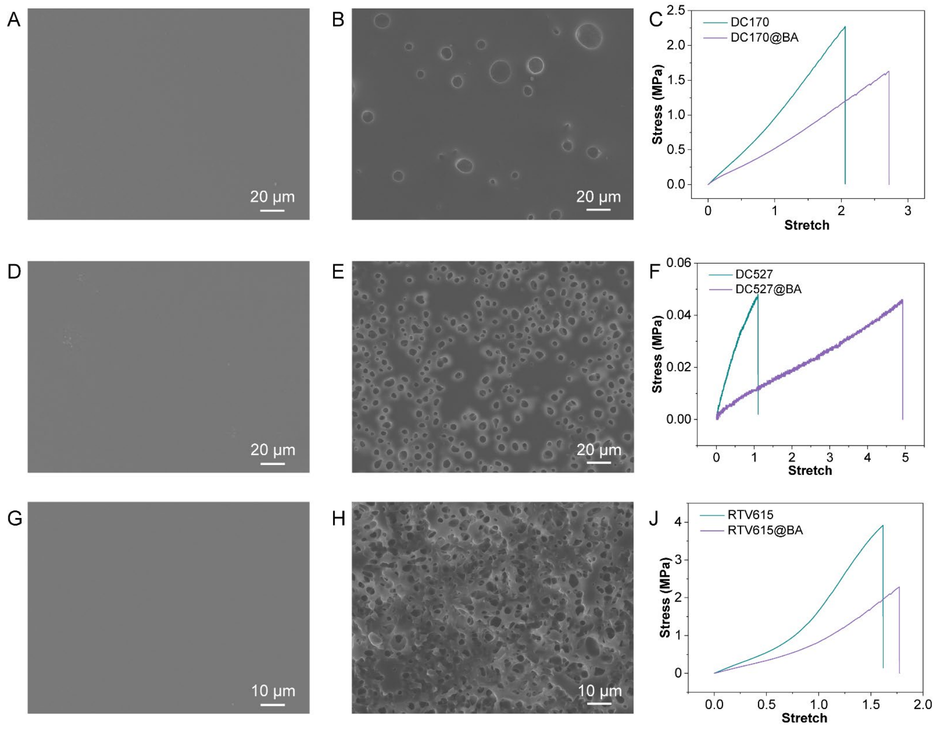

Supplementary Fig. 46. The surface SEM images of (A) DC170, (B) DC170@BA, (D) DC527, (E) DC527@BA, (G) RTV615, and (H) RTV 615@BA. Stress-stretch curves of (C) DC170 and DC170@BA, (F) DC527 and DC527@BA, (J) RTV615 and RTV615@BA.

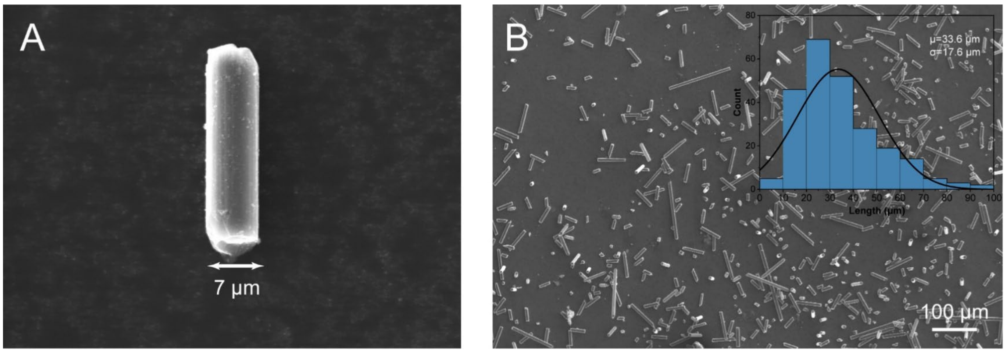

Supplementary Fig. 47. (A) Single carbon fiber (B) Distribution of carbon fiber length.

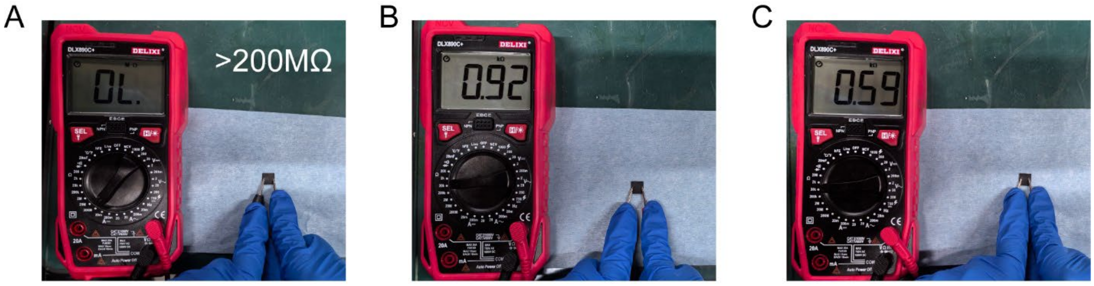

Supplementary Fig. 48. Resistance of PDMS/C, HNP-PDMS@C/-0.0, and HNP-PDMS@C/-0.1 (Sample size: 10 mm×10 mm×1 mm).

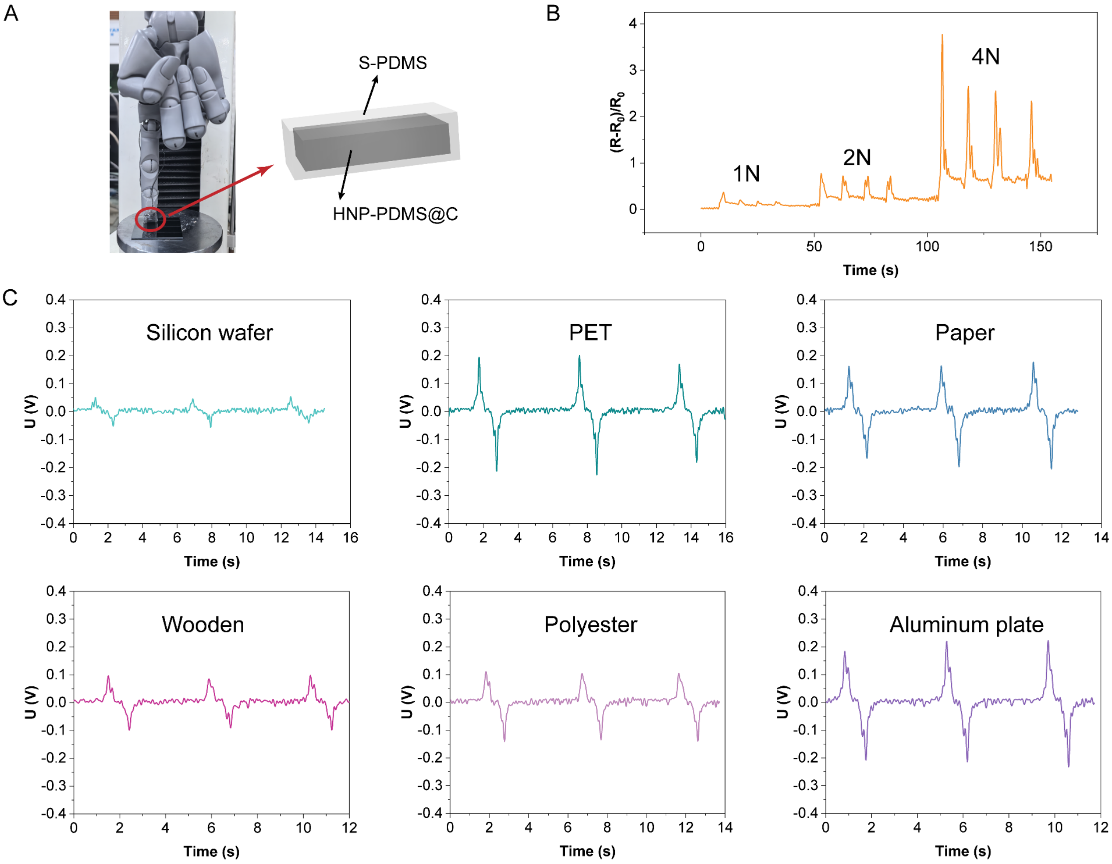

Supplementary Fig. 49. (A) Actual picture of the bionic robot hand contacting the material. (B) Resistance changes under different forces (R_0 and R are the initial resistance and the resistance under pressure, contact speed: 50 mm/min). (C) Output voltage (U) curves of 6 different materials under the same conditions (Contact speed: 500 mm/min).

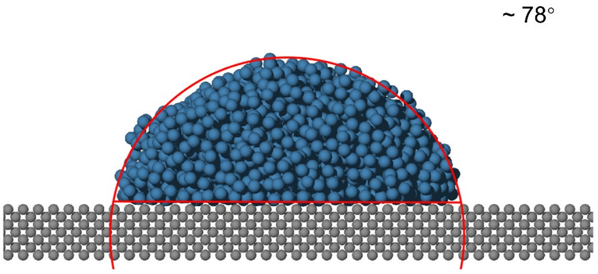

Supplementary Fig. 50. The wetting status of water on Fe (001) surface.

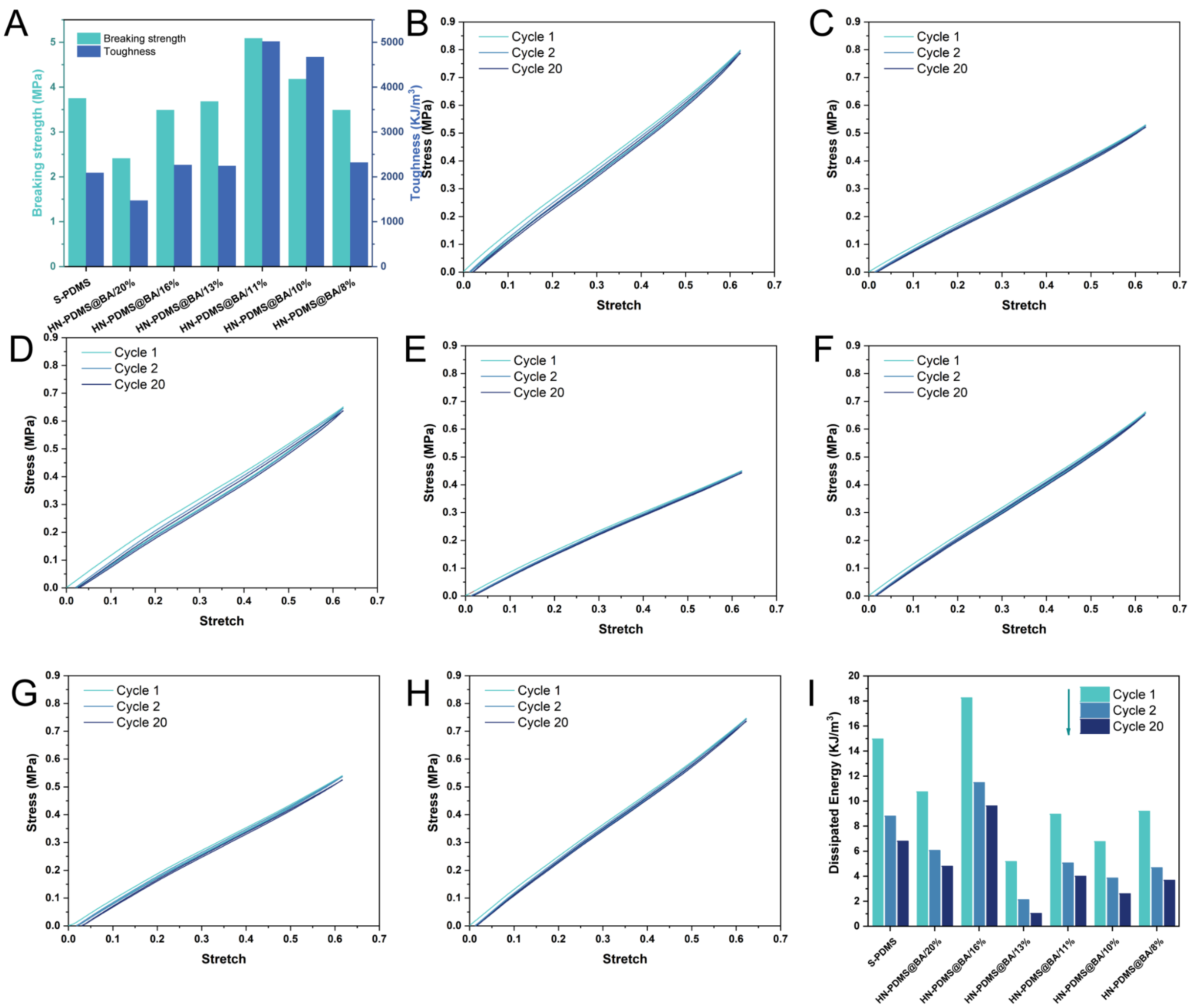

Supplementary Fig. 51. (A) Breaking strength and toughness of S-PDMS and different HN-PDMS@BA. (B)-(H) The cyclic tension conditions of S-PDMS, HN-PDMS@BA/20%, HN-PDMS@BA/16%, HN-PDMS@BA/13%, HN-PDMS@BA/11%, HN-PDMS@BA/10%, and HN-PDMS@BA/8%. (I) The dissipated energy of S-PDMS and different HN-PDMS@BA.

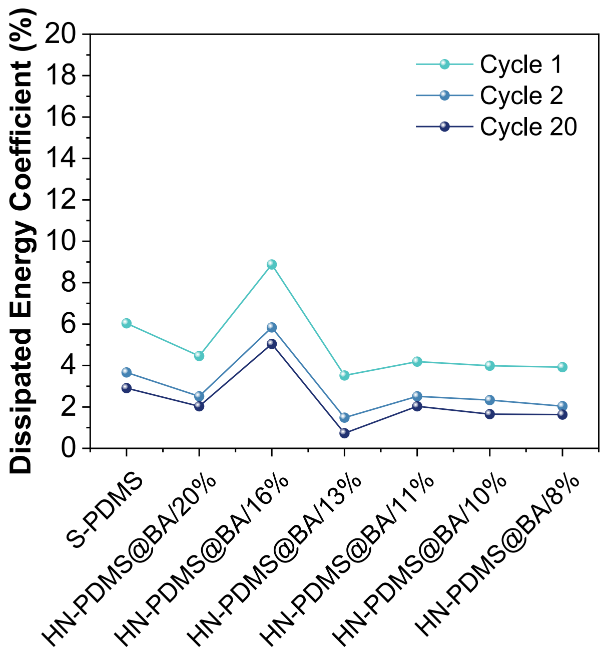

Supplementary Fig. 52. The energy dissipation coefficient of S-PDMS and different HN-PDMS@BA.

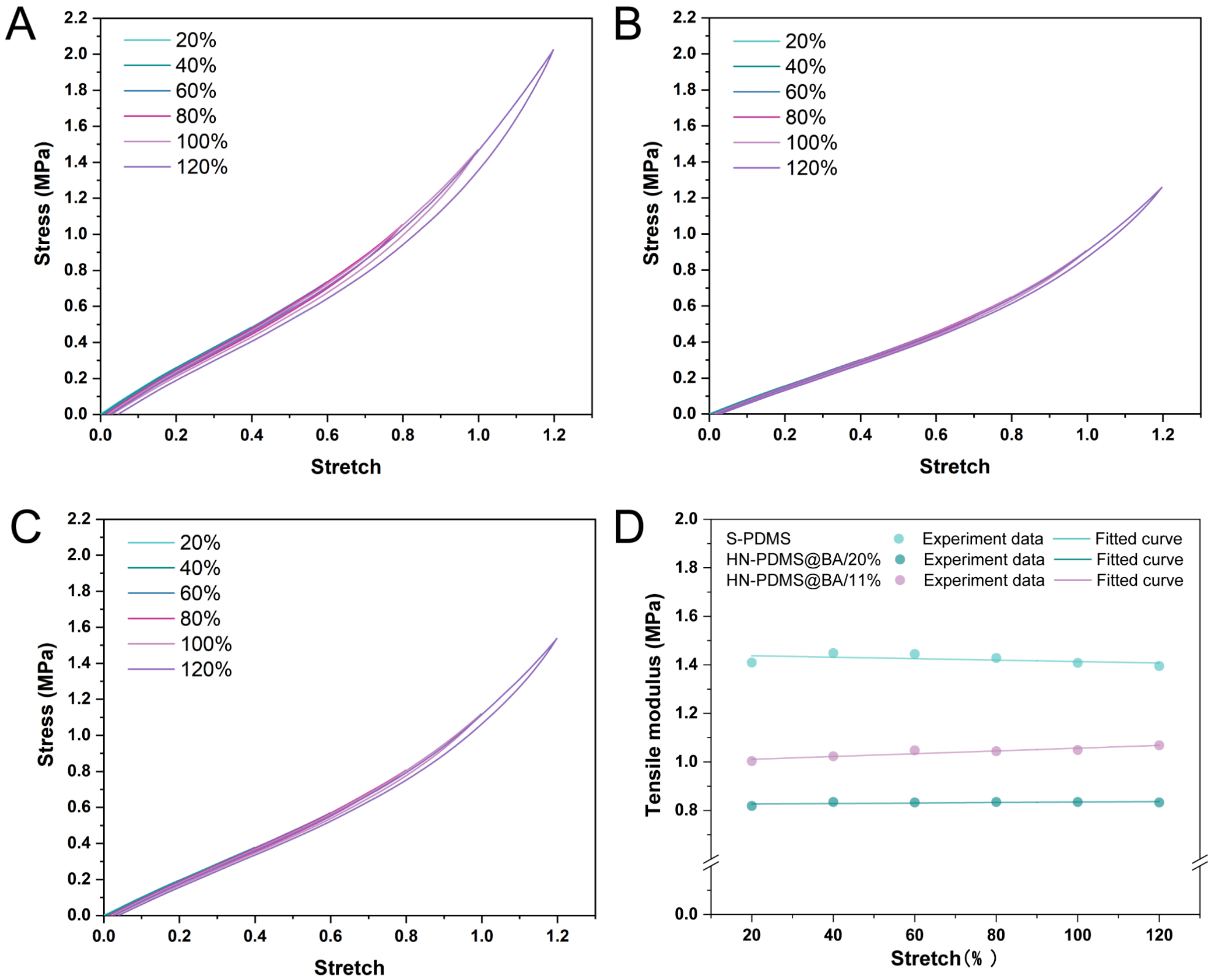

Supplementary Fig. 53. (A)-(C) Step cyclic stretching from 20% - 120% of S-PDMS, HN-PDMS@BA/20%, and HN-PDMS@BA/11%. (D) The change of elastomer's tensile modulus during step cyclic stretching.

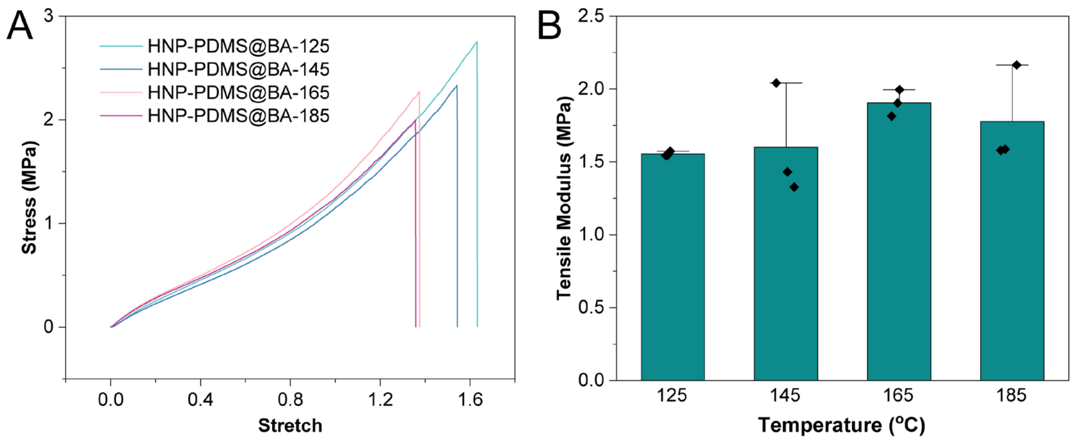

Supplementary Fig. 54. (A) Stress-stretch curves and (A) tensile modulus of HNP-PDMS@BA cured at 125 °C ~ 185 °C under -0.1 MPa. The error bars show the standard error of the mean, computed from three samples.

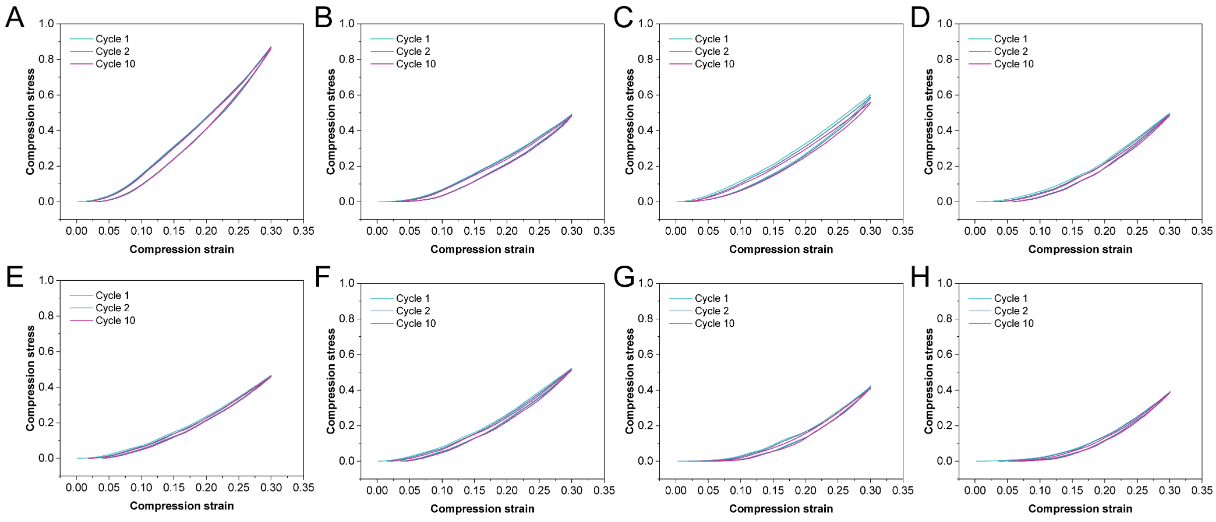

Supplementary Fig. 55. The cyclic compression curves of (A) S-PDMS, (B) HNP-PDMS@BA-125/-0.1MPa, (C) HNP-PDMS@BA-145/-0.1MPa, (D) HNP-PDMS@BA-165/-0.1MPa, (E) HNP-PDMS@BA-185/-0.1MPa, (F) HNP-PDMS@BA-125/-0.0MPa (G) PDMS@PdBA-125/-0.1MPa, and (H) PDMS@bPdBA -125/-0.1MPa.

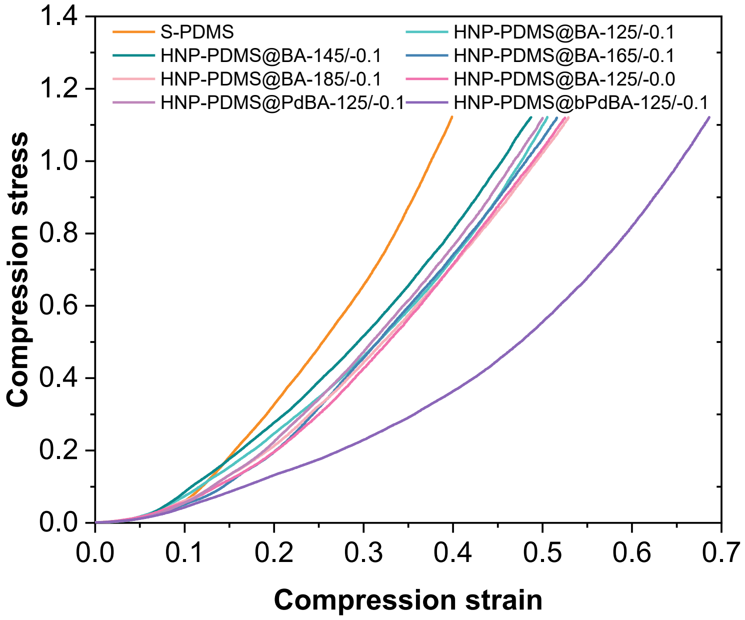

Supplementary Fig. 56. The stress-compression strain curves of different elastomers.

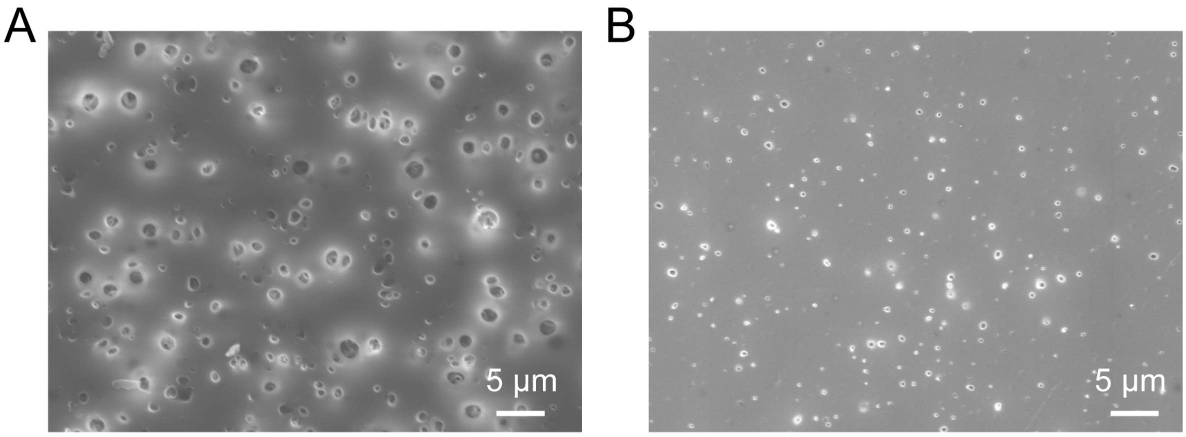

Supplementary Fig. 57. The SEM images of (A) PDMS@PdBA-125/-0.1MPa and (B) PDMS@bPdBA-125/-0.1MPa.

4. Supplementary References

1. Smith, M. K. & Northrop, B. H. Vibrational Properties of Boroxine Anhydride and Boronate Ester Materials: Model Systems for the Diagnostic Characterization of Covalent Organic Frameworks. *Chem. Mater.* **26**, 3781–3795 (2014).
2. Liu, J., Cao, D. & Zhang, L. Molecular Dynamics Study on Nanoparticle Diffusion in Polymer Melts: A Test of the Stokes−Einstein Law. *J. Phys. Chem. C* **112**, 6653–6661 (2008).
3. Matsuoka, H., Schwahn, D. & Ise, N. Observation of cluster formation in polyelectrolyte solutions by small-angle neutron scattering. 1. A steep upturn of the scattering curves from solutions of sodium poly(styrenesulfonate) at scattering vectors below 0.01 .ANG.-1. *Macromolecules* **24**, 4227–4228 (1991).
4. Kratky, O. & Porod, G. Diffuse small-angle scattering of x-rays in colloid systems. *Journal of Colloid Science* **4**, 35–70 (1949).
5. Rambo, R. P. & Tainer, J. A. Characterizing flexible and intrinsically unstructured biological macromolecules by SAS using the Porod-Debye law. *Biopolymers* **95**, 559–571 (2011).
6. Koch, M. H. J., Vachette, P. & Svergun, D. I. Small-angle scattering: a view on the properties, structures and structural changes of biological macromolecules in solution. *Quarterly Reviews of Biophysics* **36**, 147–227 (2003).
7. Järnström, J., Ihalainen, P., Backfolk, K. & Peltonen, J. Roughness of pigment coatings and its influence on gloss. *Applied Surface Science* **254**, 5741–5749 (2008).
8. Kong, D. *et al.* Control of Polymer Properties by Entanglement: A Review. *Macro Materials & Eng* **306**, 2100536 (2021).
9. Shen, J., Lin, X., Liu, J. & Li, X. Effects of Cross-Link Density and Distribution on Static and Dynamic Properties of Chemically Cross-Linked Polymers. *Macromolecules* **52**, 121–

134 (2019).
