# Supplementary material for: Borate driven heterogeneous networks for porous elastomers with improved tribological and mechanical performances
Source: Nat Commun. 2025 Dec 17;16:11183. doi: 10.1038/s41467-025-66156-2 (PMC12712056; doi:10.1038/s41467-025-66156-2)
Supplement: Supplementary file 2 — Description of Additional Supplementary Files [file 41467_2025_66156_MOESM2_ESM.pdf]

Description of Additional Supplementary Files:

Supplementary Movie 1: Water interacting with a steel ball and a hydrophobic surface.

Supplementary Movie 2: Water interacting with a steel ball and a hydrophilic surface.
